# Concomitant genetic ablation of L-type Ca_v_1.3 (α_1D_) and T-type Ca_v_3.1 (α_1G_) Ca^2+^ channels disrupts heart automaticity

**DOI:** 10.1038/s41598-020-76049-7

**Published:** 2020-11-03

**Authors:** Matthias Baudot, Eleonora Torre, Isabelle Bidaud, Julien Louradour, Angelo G. Torrente, Lucile Fossier, Leïla Talssi, Joël Nargeot, Stéphanie Barrère-Lemaire, Pietro Mesirca, Matteo E. Mangoni

**Affiliations:** 1 Institut de Génomique Fonctionnelle, Université de Montpellier, CNRS, Inserm, 141, rue de la cardonille, 34094 Montpellier, France; 2 LabEx ICST, Montpellier, France; 3 Department of Biotechnology and Biosciences, Università Degli Studi di Milano-Bicocca, Milan, Italy

**Keywords:** Physiology, Cardiology

## Abstract

Cardiac automaticity is set by pacemaker activity of the sinus node (SAN). In addition to the ubiquitously expressed cardiac voltage-gated L-type Ca_v_1.2 Ca^2+^ channel isoform, pacemaker cells within the SAN and the atrioventricular node co-express voltage-gated L-type Ca_v_1.3 and T-type Ca_v_3.1 Ca^2+^ channels (SAN-VGCCs). The role of SAN-VGCCs in automaticity is incompletely understood. We used knockout mice carrying individual genetic ablation of Ca_v_1.3 (*Ca*_*v*_*1.3*^*−/*−^) or Ca_v_3.1 (*Ca*_*v*_*3.1*^*−/*−^) channels and double mutant *Ca*_*v*_*1.3*^*−/*−^/*Ca*_*v*_*3.1*^*−/*−^ mice expressing only Ca_v_1.2 channels. We show that concomitant loss of SAN-VGCCs prevents physiological SAN automaticity, blocks impulse conduction and compromises ventricular rhythmicity. Coexpression of SAN-VGCCs is necessary for impulse formation in the central SAN. In mice lacking SAN-VGCCs, residual pacemaker activity is predominantly generated in peripheral nodal and extranodal sites by f-channels and TTX-sensitive Na^+^ channels. In beating SAN cells, ablation of SAN-VGCCs disrupted late diastolic local intracellular Ca^2+^ release, which demonstrates an important role for these channels in supporting the sarcoplasmic reticulum based “*Ca*^*2*+^ *clock*” mechanism during normal pacemaking. These data implicate an underappreciated role for co-expression of SAN-VGCCs in heart automaticity and define an integral role for these channels in mechanisms that control the heartbeat.

## Introduction

Heart automaticity is reliant on the primary pacemaker activity of the sino-atrial node (SAN) and impulse conduction through the principal components of the conduction system: the atrioventricular node (AVN), the His bundle and the Purkinje fibres network^1^. Under physiological conditions, the SAN generates cardiac automaticity whereas the AVN and the Purkinje fibres network initiate automaticity in case of SAN failure. Automaticity in cardiac cells is due to diastolic depolarization, a slow phase of the action potential cycle driving the membrane voltage from the end of the repolarization to the threshold of the following action potential^2^. While several aspects of SAN pacemaker mechanisms are still incompletely understood, there is general agreement that diastolic depolarization is generated by an interplay between the activity of ion channels of the plasma membrane and local diastolic intracellular Ca^2+^ release (LCR) from ryanodine receptors (RyRs) of the sarcoplasmic reticulum (SR)^3–5^.

Among plasmalemmal ion channels, hyperpolarization-activated “funny” f-channels of the HCN family^6^, voltage-gated Ca^2+^ channels (VGCCs)^7^, voltage-dependent tetrodotoxin (TTX)-sensitive Na^+^ channels mediating the neuronal Na^+^ current (*I*_*Na(TTX*)_)^8–11^ and transient receptor potential (TRPM and TRPC)^12–14^ channels have been shown to contribute to automaticity in the SAN or in the AVN. The contribution of RyR-dependent Ca^2+^ release to automaticity is currently interpreted in the framework of the “*coupled clock*” model of pacemaking^3,15,16^. Two functional components form the *coupled clock*: the spontaneous SR-based “*Ca*^*2*+^ *clock*” mechanism and the “*membrane clock*” (or voltage clock)^15^. A cyclical phenomenon of spontaneous voltage-independent LCR events generates the *Ca*^*2*+^ *clock*. Indeed, spontaneous LCRs can be observed in the absence of changes in membrane voltage and constitute the hallmark of the *Ca*^*2+*^ *clock*^17^. During pacemaking, spontaneous LCRs are generated by early diastolic openings of RyRs that activate the cardiac Na^+^–Ca^2+^ exchanger (NCX1)^17,18^. This electrogenic transport generates an inward current that contributes to diastolic depolarization^19^. Sequential and cyclical activation of plasmalemmal ion channels during pacemaking forms the *membrane clock*^15,16^. In the *coupled-clock* model of pacemaking, f-channels are important activators of the *membrane clock* at the beginning of diastolic depolarization^15^. In the last fraction of diastolic depolarization, T- and L-type Ca^2+^ channels activate in cooperation with NCX to drive the membrane voltage to the action potential threshold^16^. During the action potential phase, Ca^2+^ entry via L-type VGCCs refills the SR Ca^2+^ load, allowing new diastolic LCRs to occur in the following cycle. Therefore, under normal conditions, the *Ca*^*2*+^ *clock* and the *membrane clock* mutually entrain and synchronize to generate robust physiological pacemaking^15,16^ (see Refs.^3–5^ for review).

The adult mammalian heart expresses three VGCCs isoforms^7^: L-type Ca_v_1.2 and Ca_v_1.3 channels, as well as T-type Ca_v_3.1 channels. These isoforms show differential expression in the working myocardium and in automatic tissue. In particular, adult ventricular myocytes express L-type Ca_v_1.2 channels^20^, which couple excitation to contraction in the myocardium^21^. Ca_v_1.2 channels are also ubiquitously expressed in the SAN region and in the conduction system^20^. In contrast, L-type Ca_v_1.3 channels are highly expressed in automatic tissue^20^ and, in lower densities, in atria^20,22^. Finally, T-type Ca_v_3.1 channels are not functionally expressed in the adult working myocardium but show strong expression in the SAN and in the AVN^23^. Ca_v_1.3 functionally differs from the other cardiac L-type isoform. Under basal conditions, the threshold for activation of Ca_v_1.3-mediated L-type Ca^2+^ current (*I*_*CaL*_) in SAN cells is significantly more negative (− 50 mV) than that of Ca_v_1.2-mediated *I*_*CaL*_ (− 30 mV)^24^, which controls the excitation–contraction coupling mechanism in the working myocardium. β-adrenergic activation shifts the threshold of both Ca_v_1.3- and Ca_v_1.2-mediated *I*_*CaL*_ by ~ 5 mV to more negative voltages^24^. T-type Ca_v_3.1 channels activate at more negative voltages than Ca_v_1.3. In SAN cells, the threshold for activation of Ca_v_3.1-mediated T-type Ca^2+^ current (*I*_*CaT*_) is more negative than that of Ca_v_1.3-mediated *I*_*CaL*_ (− 55 mV) and its half-inactivation voltage is more negative (− 70 mV)^23,24^. Previous studies showed that L-type Ca_v_1.3 channels contribute to the generation of diastolic depolarization in the SAN^24^ and AVN^25,26^. In addition, we have showed that Ca_v_1.3 channels contribute to the generation of diastolic LCRs and are necessary to synchronize LCRs events under β-adrenergic activation of pacemaking^27^. T-type Ca_v_3.1 channels have also been shown to contribute to automaticity in the SAN^24,28^ and in the AVN^25^. Importantly, loss-of-function of Ca_v_1.3 and Ca_v_3.1 channels underlies congenital^29^ and autoimmune^30^ forms of SAN dysfunction associated with disturbances of atrioventricular conduction.

Despite this physiological relevance, it is currently unknown whether normal automaticity, heart rate and rhythm are contingent on the co-expression of L-type Ca_v_1.3 and T-type Ca_v_3.1 channels (hereby named SAN-VGCCs). In addition, genetic and functional evidence of the impact of co-expression of SAN-VGCCs within the *coupled clock* model are currently lacking. Here we hypothesised that co-expression of SAN-VGCCs is necessary to generate normal cardiac automaticity and to ensure synchrony between atrial and ventricular rhythmicity. We also hypothesised that, because of their negative voltage threshold for activation, SAN-VGCCs may constitute an important factor for generating the *coupled clock* pacemaker mechanism. To address this, we created a mouse model in which the two SAN-VGCCs expressed in the SAN and the AVN but not in the ventricle have been ablated (*Ca*_*v*_*1.3*^*−/*−^*/Ca*_*v*_*3.1*^*−/*−^). We demonstrate that co-expression of SAN-VGCCs is necessary to maintain pacemaking in the SAN and for proper impulse propagation, as well as to maintain normal impulse conduction and ventricular rhythmicity. We also show that SAN-VGCCs are necessary to generate late diastolic LCRs involved in the *coupled clock* pacemaker mechanism, a function that Ca_v_1.2 channels cannot maintain in the absence of SAN-VGCCs.

## Results

### Concomitant ablation of SAN-VGCCs abolishes VGCCs-mediated Ca^2+^ current in the voltage range of diastolic depolarization

**Figure 1:**
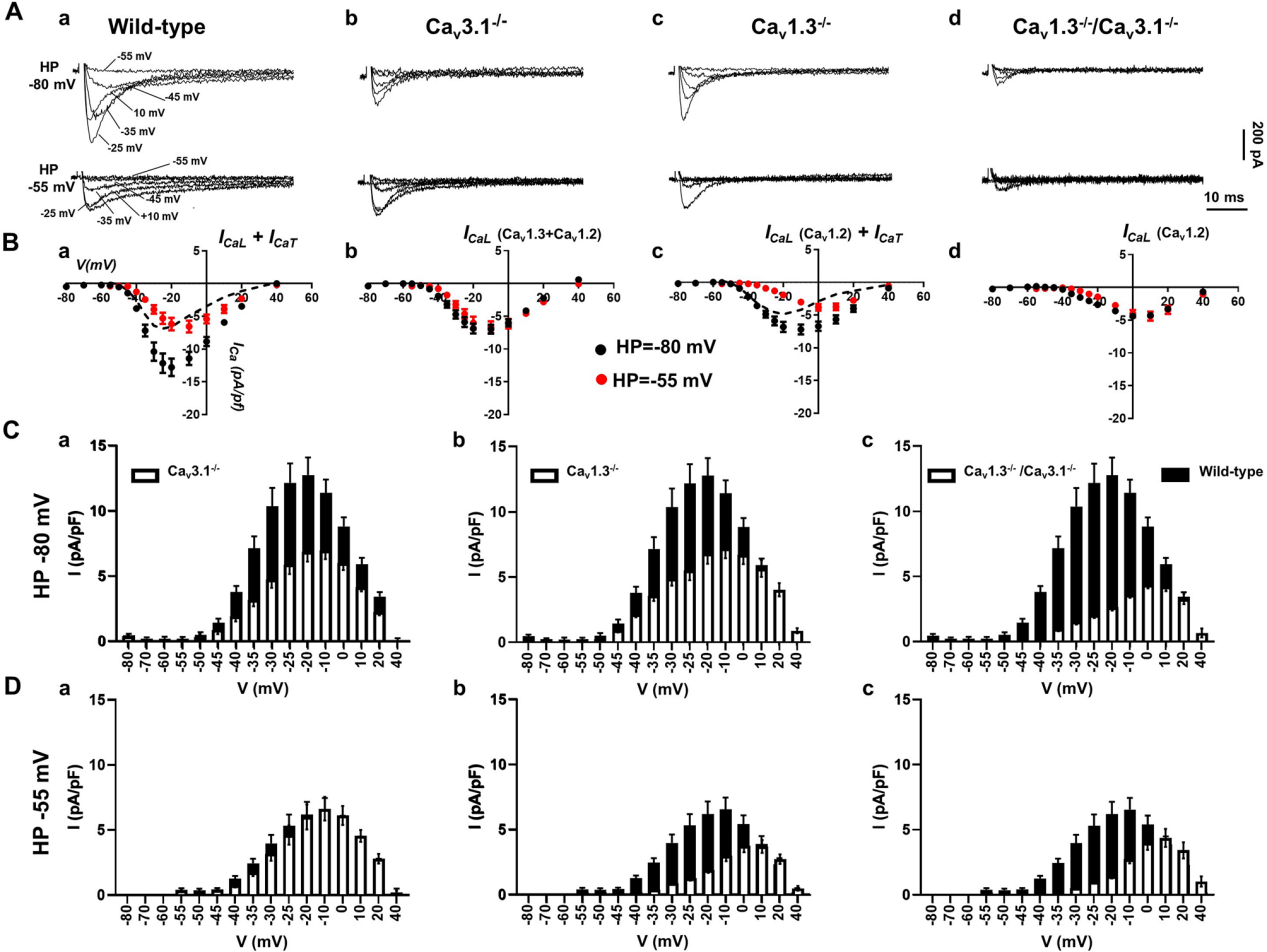
*I*_*Ca*_ in isolated SAN cells from wild-type and mutant mice. (**A**) Sample traces recorded from a HP of − 80 mV (top line) or from − 55 mV (bottom line) in SAN cells of wild-type *(WT)* (a), *Ca*_*v*_*3.1*^*−/*−^ (b), *Ca*_*v*_*1.3*^*−/*−^ (c) and *Ca*_*v*_*1.3*^*−/*−^*/Ca*_*v*_*3.1*^*−/*−^ mice (d). (**B**) Current-to-voltage (I–V) relationship of Ca^2+^ current recorded from a HP = − 80 mV (black circles) or from HP = − 55 mV (red circles) in SAN cells from the following genotypes: wild-type (a, n = 16), *Ca*_*v*_*3.1*^*−/*−^ (b, n = 15), *Ca*_*v*_*1.3*^*−/*−^ (c, n = 15) and *Ca*_*v*_*1.3*^*−/*−^*/Ca*_*v*_*3.1*^*−/*−^ mice (d, n = 18). In (a) and (b), the dashed line indicates the net *I*_CaT_ I–V curve, calculated as the difference between values obtained from HP of − 55 mV from those from HP − 80 mV. (**C**,**D**) Relative density of *I*_CaT_ and *I*_CaL_ recorded in SAN cells isolated from wild-type (black bars, n = 16), *Ca*_*v*_*3.1*^*−/*−^ (a, open bars, n = 15), *Ca*_*v*_*1.3*^*−/*−^ (b, open bars, n = 15) and *Ca*_*v*_*1.3*^*−/*−^*/Ca*_*v*_*3.1*^*−/*−^ mice (c, open bars, n = 18) at different test potentials from HP = − 80 mV (**C**) and HP = − 55 mV (**D**).

We studied the consequences of concomitant ablation of SAN-VGCCs on total VGCCs-mediated current (*I*_*Ca*_) in isolated SAN pacemaker cells from control and mutant (*Ca*_*v*_*3.1*^*−/*−^, *Ca*_*v*_*1.3*^*−/*−^ and *Ca*_*v*_*1.3*^*−/*−^*/Ca*_*v*_*3.1*^*−/*−^) mice. *I*_*Ca*_ was first recorded from a holding potential (HP) of − 80 mV to record *I*_*CaL*_ and *I*_*CaT*_^23,24^. Samples traces of total Ca^2+^ current (*I*_*Ca*_) are reported in Fig. 1A for wild-type, *Ca*_*v*_*3.1*^*−/*−^, *Ca*_*v*_*1.3*^*−/*−^ and *Ca*_*v*_*1.3*^*−/*−^*/Ca*_*v*_*3.1*^*−/*−^ SAN cells. To separate *I*_*CaL*_ from *I*_*CaT*_, we switched the HP to − 55 mV to inactivate *I*_*CaT*_ (Fig. 1A). This HP inactivates about 15–20% of Ca_v_1.3-mediated *I*_*CaL*_, but completely inactivates Ca_v_3.1-mediated *I*_*CaT*_^7^. In wild-type SAN cells, net *I*_*CaT*_ was calculated as the difference between traces obtained at HP of − 55 mV from those at HP − 80 mV (Fig. 1B-a, dashed line). We recorded only *I*_*CaL*_ flowing through Ca_v_1.3 and Ca_v_1.2 isoforms in *Ca*_*v*_*3.1*^*−/*−^ SAN cells, whereas *I*_*CaT*_ was undetectable (Fig. 1B-b). In *Ca*_*v*_*1.3*^*−/*−^ SAN cells, total *I*_*Ca*_ (black dots, Fig. 1B-c) was generated by Ca_v_3.1-mediated *I*_*CaT*_ and Ca_v_1.2-mediated *I*_*CaL*_ (red dots, Fig. 1B-c). We quantified *I*_*CaT*_ by subtracting traces obtained at the two different HPs (Fig. 1B-c, dashed line). In *Ca*_*v*_*1.3*^*−/*−^*/Ca*_*v*_*3.1*^*−/*−^ SAN cells the residual *I*_*Ca*_ recorded was Ca_v_1.2-mediated *I*_*CaL*_, which was recognizable by its activation at positive membrane voltages, and faster inactivation kinetics than Ca_v_1.3-mediated *I*_*CaL*_ recorded in wild-type cells (Fig. 1B-d). No *I*_*Ca*_ could be recorded at voltages negative to − 35 mV in *Ca*_*v*_*1.3*^*−/*−^*/Ca*_*v*_*3.1*^*−/*−^ SAN cells. A comparison between *I*_*Ca*_ densities recorded in SAN cells from wild-type and mutant animals is depicted in Fig. 1C (HP = − 80 mV) and Fig. 1D (HP = − 55 mV). In conclusion, concomitant ablation of SAN-VGCCs abolished diastolic *I*_*Ca*_ in *Ca*_*v*_*1.3*^*−/*−^*/Ca*_*v*_*3.1*^*−/*−^ SAN cells.

### Deep bradycardia and heart block in *Ca*_*v*_*1.3*^*−/*−^*/Ca*_*v*_*3.1*^*−/*−^ mice

**Figure 2:**
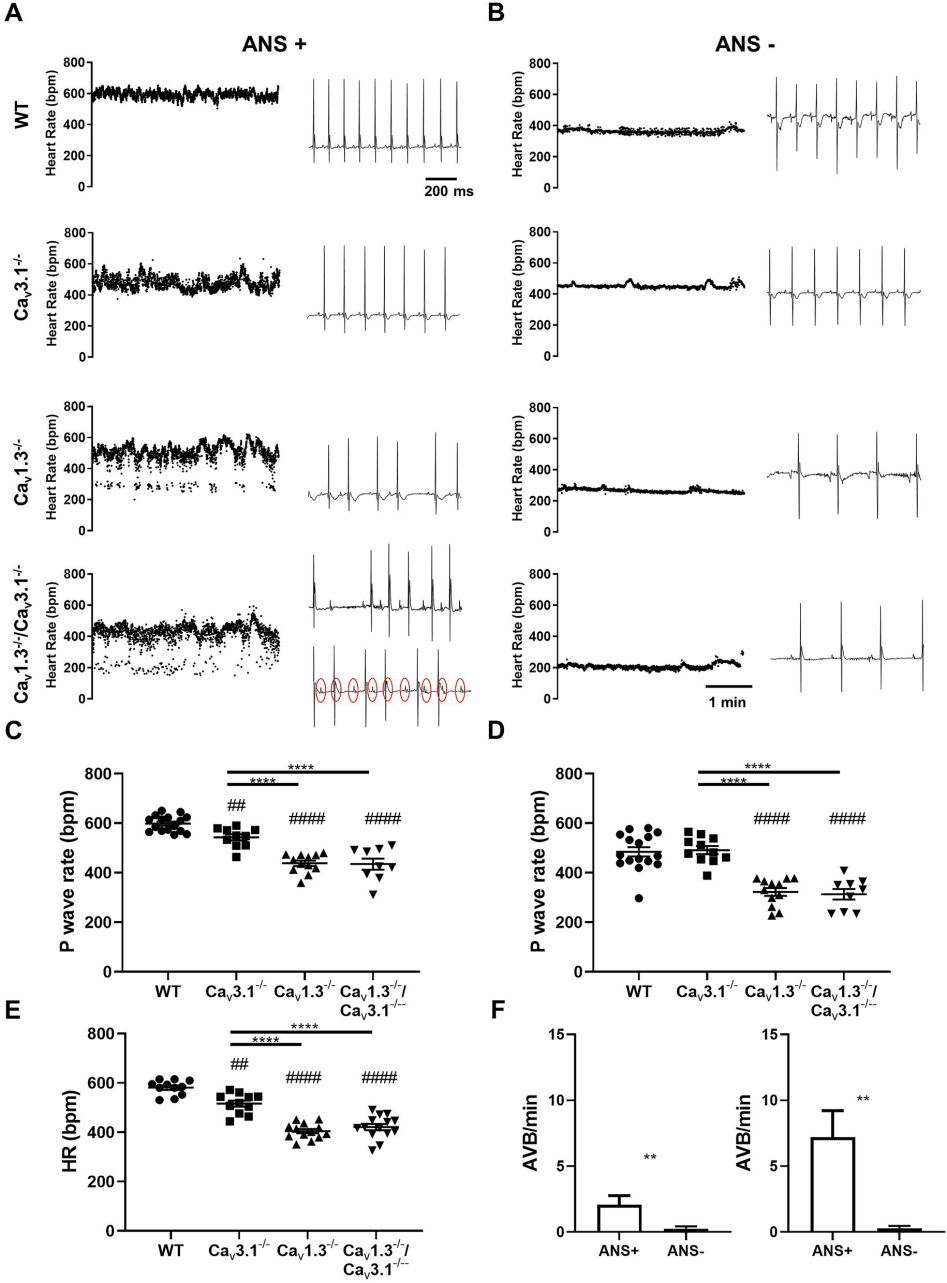
ECG recordings in wild-type and mutant mice. (**A**) Sample dot plots of heart rate (left panels) and ECG recordings (right panels) under control conditions with autonomic nervous system (ANS+) in wild-type (*WT*, *Ca*_*v*_*3.1*^*−/*−^, *Ca*_*v*_*1.3*^*−/*−^ and *Ca*_*v*_*1.3*^*−/*−^*/Ca*_*v*_*3.1*^*−/*−^ mice. Red circles indicate P waves in ECG recording in *Ca*_*v*_*1.3*^*−/−*^*/Ca*_*v*_*3.1*^*−/−*^ mice presenting 3rd degree AV block. (**B**) Dot plots of heart rate (left panels) and ECG recordings (right panels) following intraperitoneal injection of atropine (1 mg/kg) and propranolol (5 mg/kg) to inhibit the input of the autonomic nervous system (ANS−) in *WT*, *Ca*_*v*_*3.1*^*−/−*^*, Ca*_*v*_*1.3*^*−/−*^ and *Ca*_*v*_*1.3*^*−/−*^*/Ca*_*v*_*3.1*^*−/−*^mice. (**C**,**D**). Averaged rate of P waves (atrial rate) in wild-type (n = 16) and mutant mice under control conditions (ANS+) (**C**, *Ca*_*v*_*3.1*^*−/−*^ n = 10, *Ca*_*v*_*1.3*^*−/−*^ n = 12, *Ca*_*v*_*1.3*^*−/−*^*/Ca*_*v*_*3.1*^*−/−*^ n = 9) or following concomitant injection of atropine and propranolol (ANS−) (**D**, *WT* n = 16 *Ca*_*v*_*3.1*^*−/−*^ n = 11, *Ca*_*v*_*1.3*^*−/−*^ n = 12, *Ca*_*v*_*1.3*^*−/−*^*/Ca*_*v*_*3.1*^*−/−*^ n = 9) conditions. (**E**) Averaged ventricular rates (HR) in wild-type (n = 11) and mutant mice (*Ca*_*v*_*3.1*^*−/−*^ n = 11, *Ca*_*v*_*1.3*^*−/−*^ n = 13, *Ca*_*v*_*1.3*^*−/−*^*/Ca*_*v*_*3.1*^*−/*−^ n = 14) under control (ANS+) conditions. Statistics: one-way ANOVA followed by Tukey’s multiple comparisons. Whiskers indicate mean ± the SEM. (#) Indicates comparison with wild-type. (**F**) Number of atrioventricular blocks (AVB) under control and atropine and propranolol inhibition conditions in n = 12 *Ca*_*v*_*1.3*^*−/*−^ (left) and n = 9 *Ca*_*v*_*1.3*^*−/*−^*/Ca*_*v*_*3.1*^*−/*−^ (right) mice. Statistics: unpaired *t* test. (#) Indicates comparison with wild-type. Statistics: Wilcoxon matched-pairs signed rank test.

To evaluate SAN pacemaking and atrioventricular (AV) conduction, we recorded in vivo atrial rates (P–P interval) and ventricular rates (HRs) in mutant mice, under control conditions (Fig. 2A and Supplementary Fig. 1, ANS+) and following combined injection of atropine and propranolol (Fig. 2B, ANS−) to inhibit autonomic input. All mutant mice displayed significant slowing of atrial and ventricular rates: *Ca*_*v*_*3.1*^*−/*−^ (~ 10%), *Ca*_*v*_*1.3*^*−/*−^ (~ 30%) and *Ca*_*v*_*1.3*^*−/*−^*/Ca*_*v*_*3.1*^*−/*−^ (~ 35%) in comparison to wild-type counterparts under control conditions (Fig. 2A,C,E and Supplementary Fig. 1). We recorded deep bradycardia in *Ca*_*v*_*1.3*^*−/*−^ and *Ca*_*v*_*1.3*^*−/*−^*/Ca*_*v*_*3.1*^*−/*−^ mice under inhibition of autonomic nervous system (~ 310 bpm, Fig. 2B–D). The averaged atrial rates of *Ca*_*v*_*1.3*^*−/*−^ and *Ca*_*v*_*1.3*^*−/*−^*/Ca*_*v*_*3.1*^*−/*−^ mice did not differ significantly, either under control conditions or following inhibition of autonomic nervous system input, suggesting that Ca_v_3.1 channels may share a common downstream effector with Ca_v_1.3 to generate pacemaking. Both *Ca*_*v*_*1.3*^*−/*−^ and *Ca*_*v*_*1.3*^*−/*−^*/Ca*_*v*_*3.1*^*−/*−^ mice showed high incidence of 2nd degree AV block (Fig. 2A). None of the *Ca*_*v*_*1.3*^*−/*−^ mice studied presented with complete 3rd degree AV block (complete heart block). In contrast, 25% of *Ca*_*v*_*1.3*^*−/*−^*/Ca*_*v*_*3.1*^*−/*−^ mice presented complete 3rd degree AV block (Fig. 2A), indicating additive effects of SAN-VGCCs ablation on AV conduction. Inhibition of the autonomic nervous system input reduced atrial rates in all genotypes tested and abolished 2nd and 3rd degree AV blocks in mutant mice (Fig. 2F). The AV conduction time (PR interval) was increasingly prolonged in mutant *Ca*_*v*_*3.1*^*−/*−^, *Ca*_*v*_*1.3*^*−/*−^ and *Ca*_*v*_*1.3*^*−/*−^*/Ca*_*v*_*3.1*^*−/*−^ mice (Supplementary Fig. 1). The uncorrected QT interval was also significantly prolonged in *Ca*_*v*_*1.3*^*−/*−^ and *Ca*_*v*_*1.3*^*−/*−^*/Ca*_*v*_*3.1*^*−/*−^ mice (Supplementary Fig. 1). Taken together, these results indicate additive effects of Ca_v_1.3 and Ca_v_3.1 loss-of-function on AV conduction.

### Rhythm dissociation and ventricular arrhythmia in *Ca*_*v*_*1.3*^*−/*−^*/Ca*_*v*_*3.1*^*−/*−^ hearts

**Figure 3:**
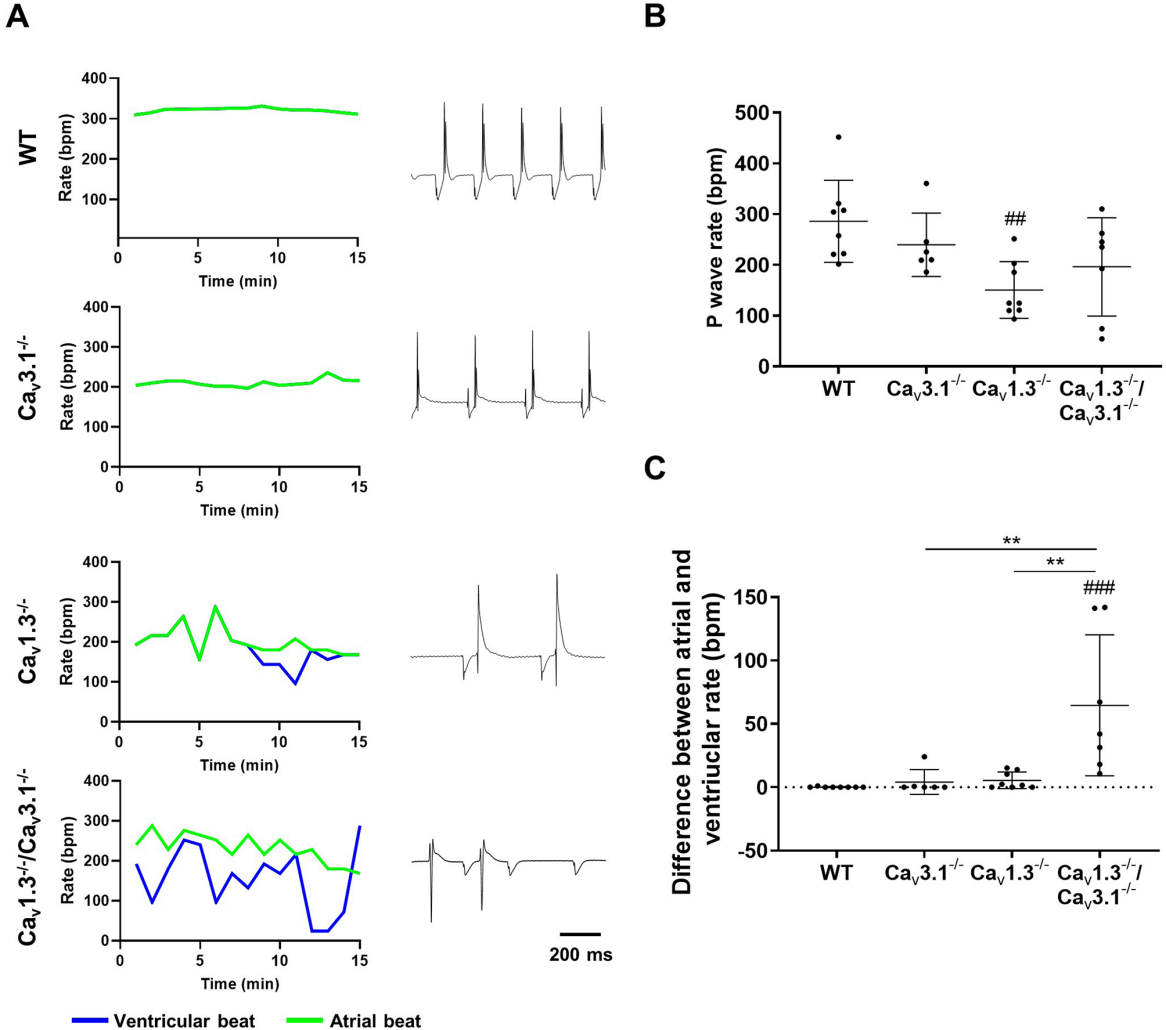
Rhythm dissociation in mutant hearts following concomitant deletion of SAN-VGCCs. (**A**) Line plots of atrial (green line) and ventricular (blue line) rates (left panel) and sample ECGs (right panel) recorded ex vivo on isolated Langendorff perfused heart under control conditions in n = 8 *WT*, n = 6 *Ca*_*v*_*3.1*^*−/*−^, n = 8 *Ca*_*v*_*1.3*^*−/*−^ and n = 7 *Ca*_*v*_*1.3*^*−/*−^*/Ca*_*v*_*3.1*^*−/*−^. (**B**) P wave rates in isolated wild-type and mutant hearts under control conditions. (**C**) Differences between atrial and ventricular rates of isolated hearts. Statistics: one-way ANOVA followed by Tukey’s multiple comparisons. Whiskers indicate mean ± the SEM. (#) Indicates comparison with wild-type.

Because we observed complete 3rd degree AV block in *Ca*_*v*_*1.3*^*−/*−^*/Ca*_*v*_*3.1*^*−/*−^ mice, we addressed the consequences of concomitant ablation of SAN-VGCCs on intrinsic heart automaticity, in the absence of autonomic input, in Langendorff-perfused hearts (Fig. 3). The spontaneous atrial rates of mutant hearts were lower than that of wild-type counterparts. Contrary to what we observed in vivo, all *Ca*_*v*_*1.3*^*−/*−^*/Ca*_*v*_*3.1*^*−/*−^ hearts investigated displayed complete 3rd degree AV block and rhythm dissociation (Fig. 3A,B). Rhythm dissociation was evidenced by the difference between measured atrial and ventricular rates in *Ca*_*v*_*1.3*^*−/*−^ and *Ca*_*v*_*1.3*^*−/*−^*/Ca*_*v*_*3.1*^*−/*−^ hearts (Fig. 3C). Furthermore, concomitant ablation of the two SAN-VGCCs was highly proarrhythmic. Indeed, *Ca*_*v*_*1.3*^*−/*−^*/Ca*_*v*_*3.1*^*−/*−^ hearts showed atrial and ventricular escape rhythms, suggesting extranodal impulse generation, as well as episodes of ventricular tachycardia (Supplementary Fig. 2). Taken together, these observations indicated that concomitant ablation of SAN-VGCCs disrupted normal automaticity of intact hearts by impairing impulse generation in the SAN, as well as by inducing complete block of AV conduction and ventricular arrhythmia.

### Concomitant ablation of SAN-VGCCs disrupts SAN automaticity and shifts the pacemaker leading sites to peripheral SAN or to extranodal locations

**Figure 4:**
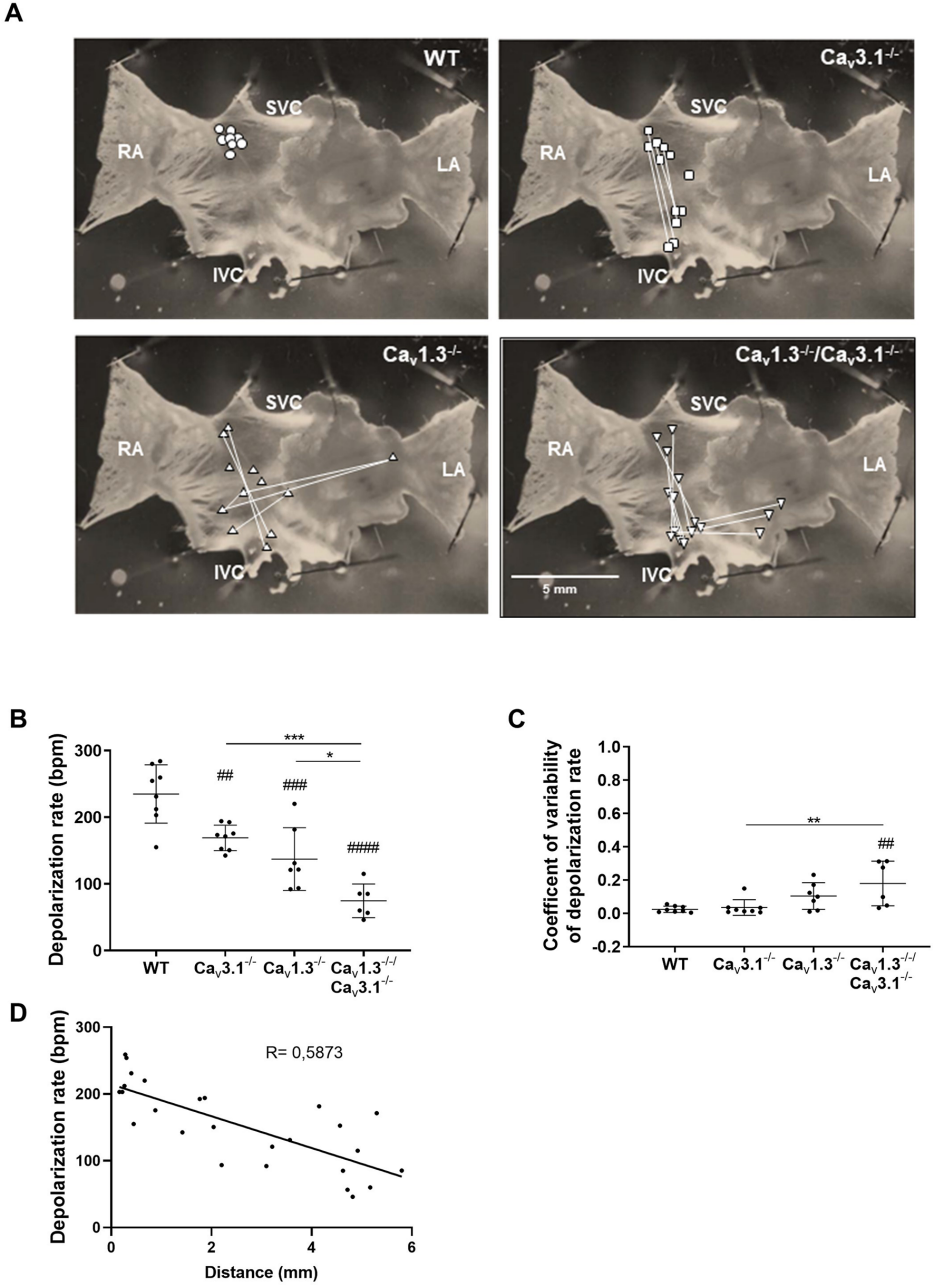
SAN automaticity and distribution of pacemaker leading sites in SAN/atria from wild-type and mutant mice. (**A**) Sample snapshots of SAN/atria preparations with points showing the position of the pacemaking leading region. Connecting lines indicate alternating leading regions in in the same mutant SAN. (**B**) Comparison between atrial rates of n = 8 wild-type, n = 8 *Ca*_*v*_*3.1*^*−/*−^*,* n = 7 *Ca*_*v*_*1.3*^*−/*−^ and n = 6 *Ca*_*v*_*1.3*^*−/*−^*/Ca*_*v*_*3.1*^*−/*−^ SAN/atria preparations. (**C**) Coefficient of variability of atrial rates from the same mice as in (**B**). Statistics: one-way ANOVA followed by Tukey’s multiple comparisons test. Whiskers indicate mean ± the SD. (**D**) Linear regression between the rate from the leading region and the distance from the normal leading region recorded in wild-type preparations. (#) Indicates comparison with wild-type.

Since we recorded limited residual SAN driven heart automaticity in isolated *Ca*_*v*_*1.3*^*−/*−^*/Ca*_*v*_*3.1*^*−/*−^ hearts we attempted to study SAN impulse formation and localization using optical mapping (OM) of membrane voltage in isolated SAN/atria preparations. All wild-type SAN/atria preparations consistently displayed the pacemaker leading site within a limited region of the central cranial side of the SAN at the entry of superior *vena cava* in the right atrium and close to the border of the *Crista Terminalis* (Fig. 4A). In contrast, we found that automaticity in *Ca*_*v*_*3.1*^*−/*−^ and *Ca*_*v*_*1.3*^*−/*−^ SAN/atria preparations was characterized by the presence of at least two leading sites with alternating dominance in 5 out of 8 and in 4 out of 7 tissues tested, respectively (Fig. 4A). In addition, 2 out of 7 *Ca*_*v*_*1.3*^*−/*−^ SAN/atria preparations showed 3 alternating leading sites. Finally, all *Ca*_*v*_*1.3*^*−/*−^*/Ca*_*v*_*3.1*^*−/*−^ preparations studied displayed alternating automaticity between two or three leading sites (Fig. 4A). In *Ca*_*v*_*3.1*^*−/*−^ preparations, secondary leading sites appeared in the correspondence of the nodal extension caudally to the inferior *vena cava*^31^. In *Ca*_*v*_*1.3*^*−/*−^ and *Ca*_*v*_*1.3*^*−/*−^*/Ca*_*v*_*3.1*^*−/*−^ SAN/atria we observed secondary leading sites also in the right or left atrium (Fig. 4A). Emergence of multiple extranodal leading sites was accompanied by increasingly slow automaticity in *Ca*_*v*_*3.1*^*−/*−^, *Ca*_*v*_*1.3*^*−/*−^ and *Ca*_*v*_*1.3*^*−/*−^*/Ca*_*v*_*3.1*^*−/*−^ SAN/atria preparations (Fig. 4B). Consistent with slowing of automaticity, the coefficient of variability of atrial rate also increased with concomitant ablation of SAN-VGCCs in comparison to wild-type counterparts (Fig. 4C). The rate of atrial impulse was negatively correlated with the distance between the alternating leading sites, suggesting increasing dominance of peripheral SAN or extranodal sites in *Ca*_*v*_*1.3*^*−/*−^*/Ca*_*v*_*3.1*^*−/*−^ SAN/atria preparations (Fig. 4D). Taken together, these observations indicate that concomitant ablation of SAN-VGCCs disrupted primary SAN automaticity and shifted pacemaker activity to peripheral SAN or to extranodal sites resulting in slow atrial rates.

### *I*_*f*_ and *I*_*NaTTX*_ sustain automaticity in *Ca*_*v*_*1.3*^−/−^ and *Ca*_*v*_*1.3*^−/−^/*Ca*_*v*_*3.1*^−/−^ mice in vivo

**Figure 5:**
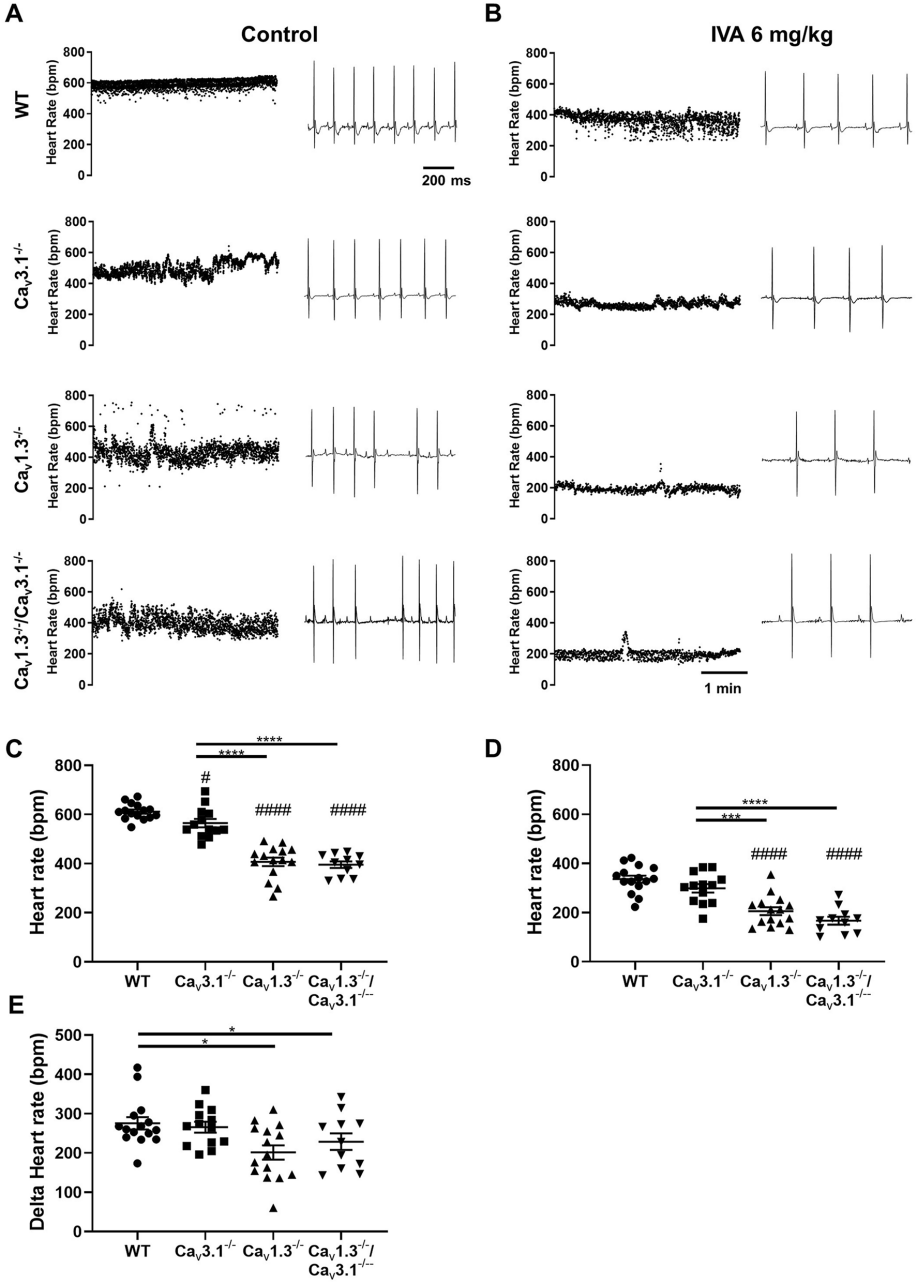
Heart rates in wild-type and mutant mice under pharmacologic inhibition of *I*_*f*_. Dot plots of heart rates and sample ECGs recorded before (**A**) and after (**B**) intraperitoneal (IP) injection of ivabradine (IVA, 6 mg/kg) in n = 14 *WT*, n = 9 *Ca*_*v*_*3.1*^*−/*−^, n = 10 *Ca*_*v*_*1.3*^*−/*−^ and n = 14 *Ca*_*v*_*1.3*^*−/*−^*/Ca*_*v*_*3.1*^*−/*−^ mice. Plotted averaged heart rates measured before (**C**) and after (**D**) IVA injection in all genotypes. (**E**) Relative effect of IVA 6 mg/kg (I.P. injection) on heart rates measured in all genotypes. In panels (**C**, **D**) and (**E**), whiskers indicate mean ± the SEM. Statistics: one-way ANOVA followed by Tukey’s multiple comparisons test. (#) Indicates comparison with wild-type mice.

Since we observed residual automaticity in hearts of *Ca*_*v*_*1.3*^*−/*−^ and *Ca*_*v*_*1.3*^*−/*−^*/Ca*_*v*_*3.1*^*−/*−^, originating either in the peripheral SAN or in extranodal regions (Fig. 4), we attempted to identify the mechanisms underlying residual pacemaking. f-(HCN) channels underlie cardiac *I*_*f*_^32,33^. Because of their direct sensitivity to intracellular cAMP, f-channels are targets of opposite regulation of HR by catecholamines and acetylcholine^2^. We thus hypothesized that the decrease in HR observed in *Ca*_*v*_*1.3*^*−/*−^ and *Ca*_*v*_*1.3*^*−/*−^*/Ca*_*v*_*3.1*^*−/*−^ mice following inhibition of the autonomic nervous system was generated by concomitant atropine induced *I*_*KACh*_ inhibition^34,35^ and propranolol induced shift of *I*_*f*_ activation to more negative voltages (Fig. 2B). We thus administered selective *I*_*f*_ blocker ivabradine (6 mg/kg) to wild-type and mutant mice and recorded HR under these conditions (Fig. 5). Ivabradine decreased the HR in all genotypes and reduced atrioventricular blocks in in *Ca*_*v*_*1.3*^*−/*−^ and *Ca*_*v*_*1.3*^*−/*−^*/Ca*_*v*_*3.1*^*−/*−^ mice (Fig. 5A–D). The HR recorded in *Ca*_*v*_*1.3*^*−/*−^*/Ca*_*v*_*3.1*^*−/*−^ mice under ivabradine administration was similar to that observed in the same mice under atropine and propranolol, suggesting that *I*_*f*_ was contributing to set the HR in *Ca*_*v*_*1.3*^*−/*−^ and *Ca*_*v*_*1.3*^*−/*−^*/Ca*_*v*_*3.1*^*−/*−^ mice in vivo under the action of the autonomic nervous system. We did not observe episodes of spontaneous atrial or ventricular arrhythmia in wild-type or mutant mice upon administration of ivabradine. The difference in HR before and after ivabradine administration was similar among wild-type and *Ca*_*v*_*3.1*^*−/*−^ mice, but was increased in *Ca*_*v*_*1.3*^*−/*−^ and *Ca*_*v*_*1.3*^*−/*−^*/Ca*_*v*_*3.1*^*−/*−^mice (Fig. 5D), showing that individual deletion of Ca_v_1.3 or concomitant ablation of SAN-VGCCs increased the relative importance of *I*_*f*_ to residual HR.

**Figure 6:**
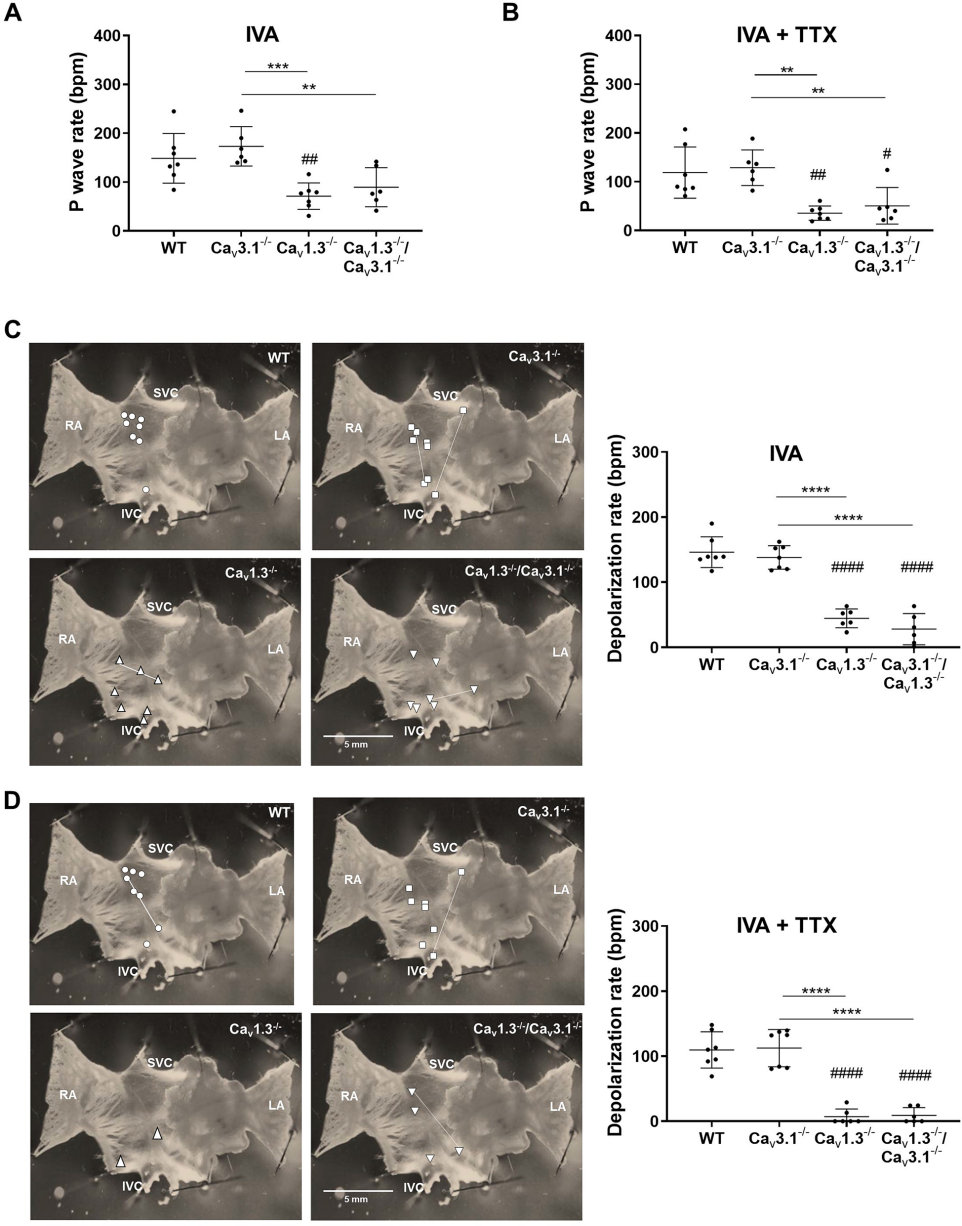
Pacemaker arrest by concomitant inhibition of *I*_*f*_ and *I*_*Na*(*TTX*)_ in *Ca*_*v*_*1.3*^*−/*−^ SAN/atria preparations. (**A**) Atrial rates of n = 7 *WT*, n = 6 *Ca*_*v*_*3.1*^*−/*−^*,* n = 7 *Ca*_*v*_*1.3*^*−/*−^ and n = 6 *Ca*_*v*_*1.3*^*−/*−^*/Ca*_*v*_*3.1*^*−/*−^ isolated Langendorff hearts under ivabradine (IVA, 10 µM) perfusion. (**B**) Atrial rates of isolated hearts under IVA 10 µM + TTX 100 nM perfusion. (**C**) Sample snapshots of the localization of leading regions under IVA 10 µM perfusion (left panels) and averaged rates of depolarization in n = 7 *WT*, n = 7 *Ca*_*v*_*3.1*^*−/*−^ , n = 6 *Ca*_*v*_*1.3*^*−/*−^ and n = 6 *Ca*_*v*_*1.3*^*−/*−^*/Ca*_*v*_*3.1*^*−/*−^ SAN/atria preparations. (**D**) Same representation as in (**C**) with leading region in IVA 10 µM + TTX 100 nM perfusion. Statistics: one-way ANOVA followed by Tukey’s multiple comparisons test. Whiskers indicate mean ± the SD. (#) Indicates comparison with wild-type.

We then investigated the origin of the residual SAN automaticity following inhibition of *I*_*f*_ under ex vivo conditions, to prevent potentially lethal bradycardia in vivo. Similarly to in vivo conditions, ivabradine (10 µM) induced low HRs in isolated hearts from mutant *Ca*_*v*_*1.3*^*−/*−^ and *Ca*_*v*_*1.3*^*−/*−^*/Ca*_*v*_*3.1*^*−/*−^ mice (< 100 bpm; Fig. 6A). Previous work showed that *I*_*Na*(*TTX*)_ contributes to the basal HR and to SAN pacemaker activity in mammals^8–11^. We thus employed a moderate concentration of TTX (100 nM) to investigate the contribution of this current to the residual automaticity of *Ca*_*v*_*1.3*^*−/*−^ and *Ca*_*v*_*1.3*^*−/*−^*/Ca*_*v*_*3.1*^*−/*−^ hearts under conditions of *I*_*f*_ block by ivabradine. TTX further reduced HR in *Ca*_*v*_*1.3*^*−/*−^ and *Ca*_*v*_*1.3*^*−/*−^*/Ca*_*v*_*3.1*^*−/*−^ hearts to very low levels (< 50 bpm; Fig. 6B). Since part of this residual automaticity could come from impulse generation in extranodal sites (Fig. 5), we directly investigated SAN automaticity by OM of the pacemaker impulse following pharmacologic inhibition of *I*_*f*_ (Fig. 6C), or concomitant *I*_*f*_ and *I*_*Na*(*TTX*)_ blockade (Fig. 6D). Blockade of *I*_*Na*(*TTX*)_ in SANs perfused with ivabradine arrested impulse generation in 4/6 *Ca*_*v*_*1.3*^*−/*−^ and 3/6 *Ca*_*v*_*1.3*^*−/*−^*/Ca*_*v*_*3.1*^*−/*−^ preparations (Fig. 6D). Very low rates of spontaneous depolarization were recorded in 5 SAN/Atria preparations from *Ca*_*v*_*1.3*^*−/*−^ and *Ca*_*v*_*1.3*^*−/*−^*/Ca*_*v*_*3.1*^*−/*−^ mice still presenting residual automaticity (< 40 bpm, Fig. 6D). In contrast, ablation of SAN-VGCCs did not affect the impulse conduction time to the atria (Supplementary Fig. 3). Similarly, ivabradine and TTX did not prolong the impulse conduction times to the right and left atria (Supplementary Fig. 3). These data indicate that while SAN-VGCCs strongly affected SAN automaticity, they were not directly involved in impulse conduction from the SAN to atria.

**Figure 7:**
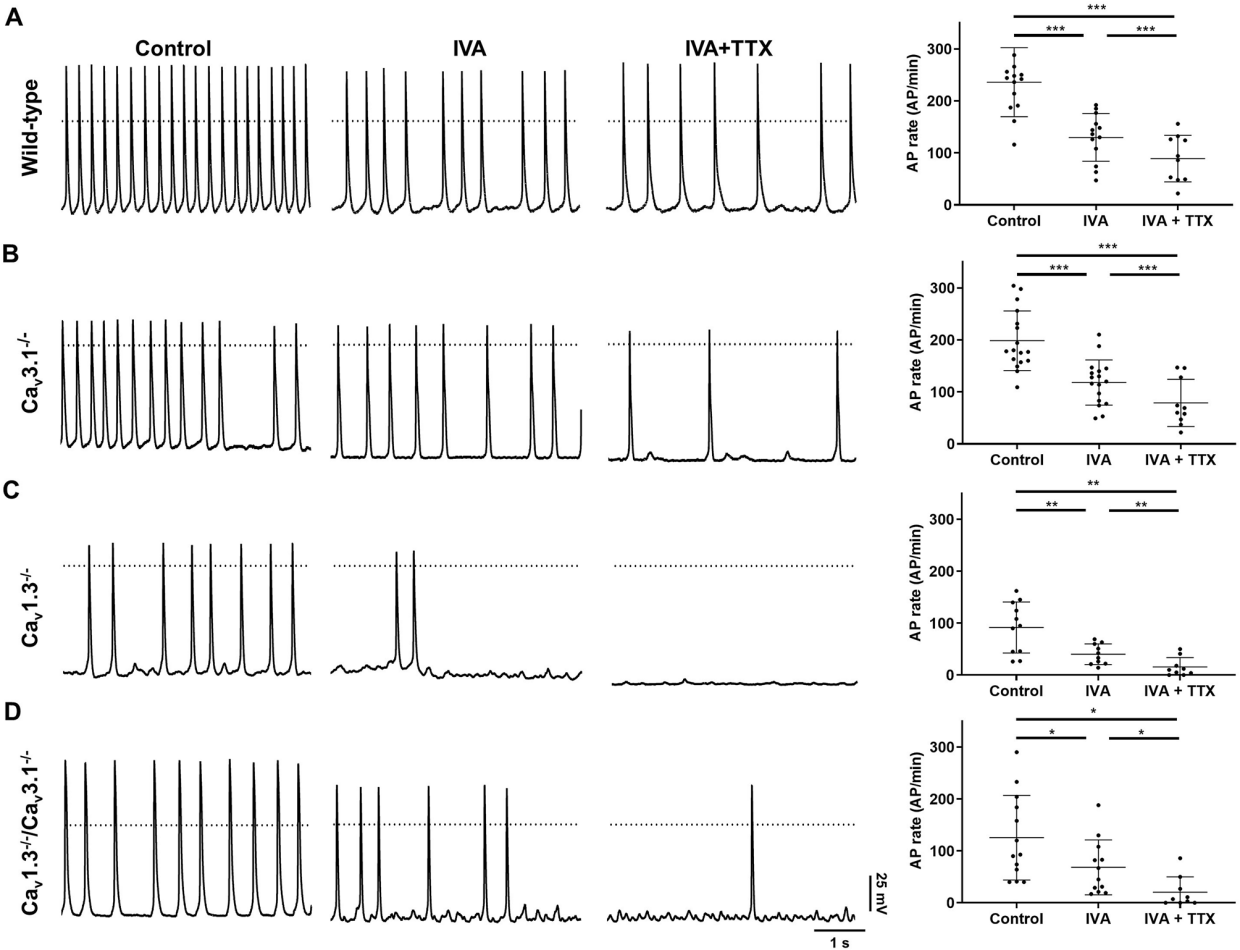
Pacemaker activity in SAN cells from wild-type and mutant mice. Sample perforated-patch action potential recordings (left panels) of SAN cells under control conditions (control), following perfusion of IVA (3 µM) and concomitant perfusion of IVA and TTX (IVA + TTX, 50 nM), from wild-type (**A**, n = 14), *Ca*_*v*_*3.1*^*−/*−^ (**B**, n = 17), *Ca*_*v*_*1.3*^*−/*−^ (**C**, n = 11) and *Ca*_*v*_*1.3*^*−/*−^*/Ca*_*v*_*3.1*^*−/*−^ (**D**, n = 13). The left panels show corresponding averaged rates of action potentials. Statistics: one-way ANOVA followed by Holm–Sidak multiple comparisons test. Whiskers indicate mean ± SD.

Concomitant ablation of SAN-VGCCs impaired the generation of SAN impulse in the SAN (Figs. 3, 4). Hence, we recorded automaticity of isolated SAN cells from wild-type and mutant mice (Fig. 7). Individual Ca_v_1.3 knockout and concomitant ablation of the two SAN-VGCCs similarly slowed basal automaticity of isolated SAN cells and reduced the slopes of linear and exponential phases of the diastolic depolarization (Fig. 7A–D, Supplementary Table 1). In addition, individual Ca_v_1.3 or concomitant SAN-VGCCs ablation hyperpolarized the action potential threshold to more negative voltages in comparison to wild-type and *Ca*_*v*_*3.1*^*−/*−^ SAN cells, without affecting the cells’ maximum diastolic potential (Supplementary Table 1). Superfusion of ivabradine alone significantly slowed automaticity of SAN cells isolated from all genotypes (Fig. 7A–D). Ivabradine also significantly hyperpolarized the maximum diastolic potential in SAN cells (Supplementary Table 2), indicating that *I*_*f*_ contributed to set the equilibrium between inward and outward currents at the end of the repolarization phase. Concomitant perfusion of ivabradine and TTX further slowed automaticity in comparison to ivabradine alone in SAN cells from all genotypes (Fig. 7A–D). Concomitant perfusion of ivabradine and TTX slowed but did not arrest automaticity of wild-type or *Ca*_*v*_*3.1*^*−/*−^ SAN cells (Fig. 7A,B). In contrast with *Ca*_*v*_*3.1*^*−/*−^ SAN cells and consistently with OM recordings, concomitant inhibition of *I*_*f*_ and *I*_*Na*(*TTX*)_ slowed the rate of spontaneous action potentials by ~ 80% and arrested automaticity in 2 out of 9 of *Ca*_*v*_*1.3*^*−/*−^ and in 3 out of 9 *Ca*_*v*_*1.3*^*−/*−^*/Ca*_*v*_*3.1*^*−/*−^ isolated SAN cells (Fig. 7C,D). We tested also if concomitant selective pharmacologic inhibition of SAN-VGCCs affected automaticity of SAN cells similarly to genetic ablation. To obtain selective inhibition of Ca_v_1.3 channels, we employed SAN cells from mice carrying dihydropyridine (DHP) resistant Ca_v_1.2 channels (*Ca*_*v*_*1.2*^*DHP−/*−^, see Ref.^36^). In *Ca*_*v*_*1.2*^*DHP−/*−^ mice, Ca_v_1.2 channels have been rendered insensitive to DHP by knock-in a point mutation in the channel DHP binding site^36^. Concomitant perfusion of the T-type selective inhibitor TTA-A_2_ and nifedipine slowed the rate of spontaneous action potentials by ~ 90% (Supplementary Fig. 4 and Supplementary Table 3). These data indicate that the strong reduction of SAN automaticity following ablation of SAN-VGCCs could not be explained by a remodeling phenomenon of the pacemaker mechanism in mice globally lacking SAN-VGCCs.

### Concomitant ablation of SAN-VGCCs reduced late diastolic local Ca^2+^ release linked to the *coupled clock* pacemaker mechanism

**Figure 8:**
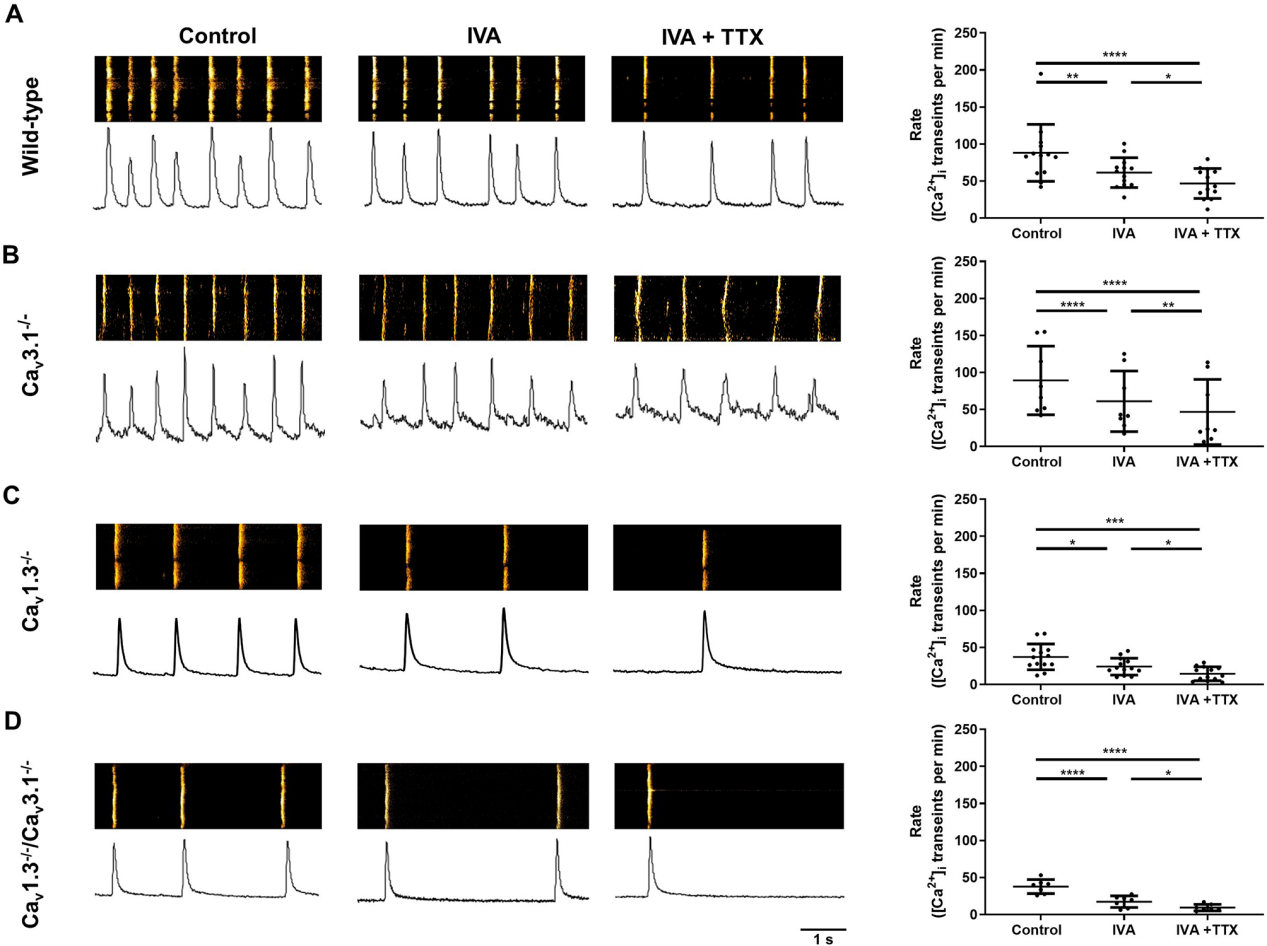
Confocal line scan imaging of intracellular Ca^2+^ ([Ca^2+^]_i_) release during pacemaker activity of SAN cells from wild-type and mutant mice. Confocal line scan (left) images (top, left), corresponding sample traces of the time integral (bottom, left) and averaged frequency of spontaneous [Ca^2+^]_i_ transients (right) of SAN cells form wild-type (**A**, n = 13), *Ca*_*v*_*3.1*^*−/*−^ (**B**, n = 8), *Ca*_*v*_*1.3*^*−/*−^ (**C**, n = 13) and *Ca*_*v*_*1.3*^*−/*−^*/Ca*_*v*_*3.1*^*−/*−^ (**D**, n = 7) loaded with Fluo-4 and perfused with Tyrode’s solution (left) or IVA 3 µM (center) or IVA 3 µM + TTX 50 nM (right). Statistics: one-way ANOVA followed by Holm–Sidak multiple comparisons test. Whiskers indicate mean ± the SD.

Experiments on SAN/Atria preparations and recordings of isolated SAN cells from *Ca*_*v*_*1.3*^*−/*−^ and *Ca*_*v*_*1.3*^*−/*−^*/Ca*_*v*_*3.1*^*−/*−^ mice under concomitant inhibition of *I*_*f*_ and *I*_*Na*(*TTX*)_ showed arrest of automaticity. This observation suggests that, following ablation of SAN-VGCCs, functional association between RyR-dependent LCRs underlying the *Ca*^*2*+^ *clock* and L-type Ca_v_1.2 channels could not consistently generate residual pacemaking under these conditions. We previously showed that Ca_v_1.3 channels positively regulate the frequency and synchrony of LCRs during pacemaking^27^. Therefore, we recorded RyR-dependent Ca^2+^ release and [Ca^2+^]_i_ dynamics in wild-type and mutant SAN cells to investigate the consequences of SAN-VGCCs ablation on late diastolic LCRs, which constitute one hallmark of the *coupled clock* pacemaker mechanism^15^. In line with recordings of spontaneous action potentials, the frequency of spontaneous [Ca^2+^]_i_ transients was similar in *Ca*_*v*_*1.3*^*−/*−^ and *Ca*_*v*_*1.3*^*−/*−^*/Ca*_*v*_*3.1*^*−/*−^ SAN cells, but lower than in wild-type and *Ca*_*v*_*3.1*^*−/*−^ counterparts (Fig. 8A–D). Concomitant blockade of *I*_*f*_ and *I*_*Na*(*TTX*)_ reduced the frequency of spontaneous [Ca^2+^]_i_ transients by 62% in *Ca*_*v*_*1.3*^*−/*−^ and by 74% in *Ca*_*v*_*1.3*^*−/*−^*/Ca*_*v*_*3.1*^*−/*−^ SAN cells, leading to very low averaged rates of automaticity (< 15 bpm; Fig. 8C,D).

**Figure 9:**
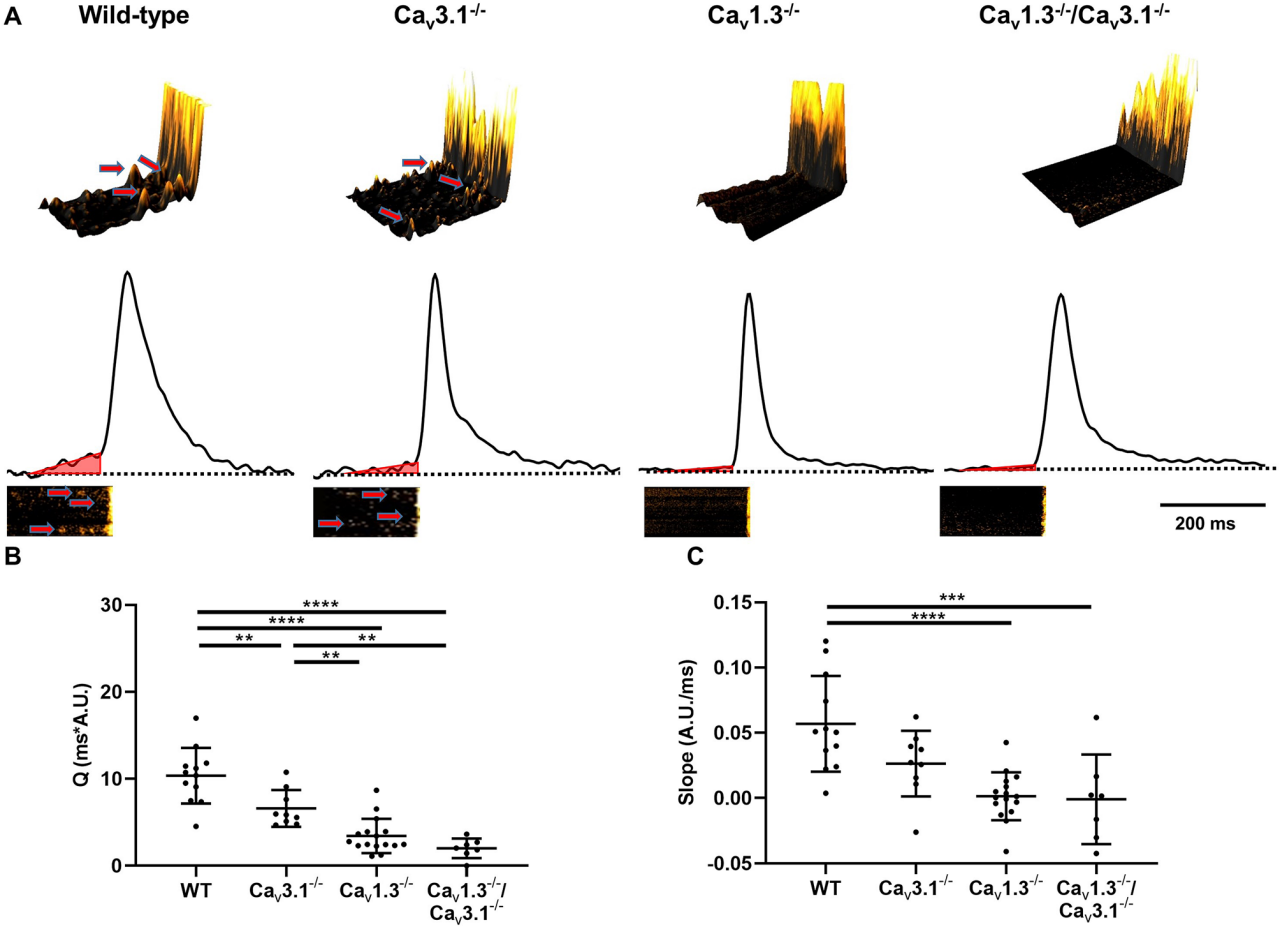
Genetic ablation of SAN-VGCCs suppresses late diastolic local [Ca^2+^]_i_ release (LCR). (**A**, top panel). Samples of 3D reconstruction of recordings of the change in fluorescence ratio F/F_0_ recorded 150 ms before the spontaneous cell-wide [Ca^2+^]_i_ release transient. Red arrows indicate late diastolic LCR. (**A**, bottom panel). Corresponding samples traces of the time integral of the fluorescence ratio F/F_0_ signal recorded from wild-type, *Ca*_*v*_*3.1*^*−/*−^, *Ca*_*v*_*1.3*^*−/*−^*, Ca*_*v*_*1.3*^*−/*−^*/Ca*_*v*_*3.1*^*−/*−^ isolated SAN cells. Red triangles represent the zone considered for calculating the slope and time integral of ramp phases, which reflects late diastolic LCR. The ramp phase was calculated starting 150 ms before the peak of the [Ca^2+^]_i_ transient. The bottom insets show corresponding 2D line scan images. Time integral (Q) of F/F_0_ (**B**) and slope (**C**) of the ramp phase in n = 12 wild-type, n = 9 *Ca*_*v*_*3.1*^*−/*−^, n = 16 *Ca*_*v*_*1.3*^*−/*−^ and n = 7 *Ca*_*v*_*1.3*^*−/*−^*/Ca*_*v*_*3.1*^*−/*−^ isolated SAN cells. A.U. indicate arbitrary units of the F/F_0_ ratio. Statistics: one-way ANOVA followed by Holm–Sidak multi comparison test.

We then analyzed LCRs events occurring in the late diastolic interval^3^. These late diastolic LCRs generate an ascending phase (ramp) of the average fluorescence ratio (F/F_0_) signal (Fig. 9A)^27,37^. We observed reduced events of late diastolic LCRs in *Ca*_*v*_*1.3*^*−/*−^ and *Ca*_*v*_*1.3*^*−/*−^*/Ca*_*v*_*3.1*^*−/*−^ SAN cells (Fig. 9A). The reduction in LCRs was reflected in diminished time integral of the ramp phase in comparison to wild-type cells (Fig. 9B). The slope of the ramp phase is indicative of the degree of synchronization of late LCRs and constitute an important parameter of the capability of the *coupled clock* to promote diastolic NCX1 activity hence pacemaking^15,16,38^. The slope of the ramp phase was strongly decreased in *Ca*_*v*_*1.3*^*−/*−^ and *Ca*_*v*_*1.3*^*−/*−^*/Ca*_*v*_*3.1*^*−/*−^ SAN cells (Fig. 9C). We did not record significant differences in the time integral or ramp slope between *Ca*_*v*_*1.3*^*−/*−^ and *Ca*_*v*_*1.3*^*−/*−^*/Ca*_*v*_*3.1*^*−/*−^ SAN cells. In conclusion, these observations indicated that concomitant ablation of SAN-VGCCs disrupted late diastolic LCRs, which constitute an important component of the *coupled clock* pacemaker mechanism.

We previously showed that ablation of Ca_v_1.3 channels preserves the SR Ca^2+^ load^27^. Therefore, we challenged wild-type and mutant SAN cells by rapid dumping of [Ca^2+^]_i_ using caffeine (10 mM). The caffeine-evoked [Ca^2+^]_i_ transient of wild-type and *Ca*_*v*_*3.1*^*−/*−^ SAN cells showed comparable amplitudes (F/F_0_, Supplementary Fig. 5A). In contrast, *Ca*_*v*_*1.3*^*−/*−^ and *Ca*_*v*_*1.3*^*−/*−^*/Ca*_*v*_*3.1*^*−/*−^ SAN cells showed increased F/F_0_ value in comparison to wild-type counterparts (Supplementary Fig. 5A). The decay time (τ) of the caffeine-evoked [Ca^2+^]_i_ transient did not differ between wild-type and mutant SAN cells, indicating similar activities of SERCA and NCX in all genotypes tested (Supplementary Fig. 5B). Finally, we compared the total number of LCRs, irrespectively from their relative position in diastolic interval, as we described previously^27^. We did not record significant differences in the total number of diastolic LCRs under perfusion of ivabradine (3 µM) or concomitant perfusion of ivabradine (3 µM) and TTX (50 nM) in comparison to control conditions in all the genotypes tested (Supplementary Fig. 5C), indicating that inhibition of *I*_*f*_ or *I*_*Na(TTX)*_ did not alter the function of RyRs. Taken together, these results show that concomitant ablation of SAN-VGCCs disrupts late diastolic LCRs underlying the SAN *coupled clock* mechanism of pacemaking.

## Discussion

### Impact of the study

In this study, we show for the first time that concomitant ablation of SAN-VGCCs disrupts heart automaticity and suppresses late diastolic LCRs, which are an important factor of the *coupled clock* pacemaker mechanism in SAN cells. SAN-VGCCs ablation compromised SAN impulse generation, impaired atrioventricular conduction and destabilized ventricular rhythmicity. Particularly, we show that Ca_v_3.1 channels play an important role in stabilizing atrial and ventricular rhythmicity in isolated *Ca*_*v*_*1.3*^*−/*−^ hearts. We also report that in both *Ca*_*v*_*1.3*^*−/*−^ and *Ca*_*v*_*1.3*^*−/*−^*/Ca*_*v*_*3.1*^*−/*−^ SAN/atria preparations, residual pacemaker activity is predominantly generated in peripheral nodal and extranodal sites by f-channels and TTX-sensitive Na^+^ channels.

The physiological role of SAN-VGCCs in orchestrating heart automaticity has been long overlooked. Therefore, designs of many currently published studies and review articles on physiopathology of heart automaticity do not still properly factor SAN-VGCCs. The original formulation of the *coupled clock* model of pacemaking ascribed predominant roles for f-channels and spontaneous voltage-independent diastolic LCRs, respectively as key components of the *membrane clock* and of the *Ca*^2+^ *clock* in the generation of the diastolic depolarization^3,5,15^. In this model, cooperation between f-channels and NCX1 was the dominant electrogenic mechanism in the generation of diastolic depolarization. The contribution of *I*_*CaT*_ was predicted to be reduced in comparison to that of *I*_*f*_ and NCX1^15^. Another important factor in the *coupled clock* is high basal intracellular cAMP concentrations and elevated activity of protein kinases that contribute to mutual entrainment between diastolic RyR-dependent Ca^2+^ release underlying the *Ca*^*2*+^ *clock* and the *membrane clock*. The earlier *coupled clock* model by Maltsev and Lakatta^15^ also included activation of *I*_*CaL*_ at positive voltages corresponding to the action potential threshold^15^. Thus, while *I*_*CaL*_ did not directly contribute to the generation of diastolic depolarization per se, Ca^2+^ entry via *I*_*CaL*_ during the action potential phase was necessary to restore SR Ca^2+^ load to prime a new cycle of diastolic LCRs^15^. However, this *coupled clock* model did not include evidence showing that SAN cells express both Ca_v_1.2-mediated *I*_*CaL*_, activating at positive systolic voltages, as well as Ca_v_1.3-mediated *I*_*CaL*_, activating at diastolic voltages and supplying Ca^2+^ entry during diastolic depolarization^24^. In addition, further work from our group showed that Ca^2+^ entry via Ca_v_1.3 channels control RyR-dependent Ca^2+^ release by triggering and synchronizing diastolic LCRs events^27^. The most recent version of the *coupled clock* model of pacemaking by Lyaskov et al.^16^ includes a more negative activation threshold for activation of *I*_*CaL*_^16^ (− 40 mV, see Ref.^39^), than in the previous version of the model^15^. Numerical simulations using the Lyaskov et al. *coupled clock* model^16^ predict that this negative shift of *I*_*CaL*_ activation induces functional cooperativity, between LCRs, NCX1 and *I*_*CaL*_ to generate exponential phase of diastolic depolarization (referred to as “ignition phase”). The present study is consistent with this model. However, and importantly for the current models of pacemaking, we now provide direct genetic and functional evidence showing that the mechanism of SAN dysfunction following ablation of SAN-VGCCs involves suppression of Ca^2+^ entry in the voltage range spanning the diastolic depolarization and impairment of generation of late diastolic LCRs. Thus, SAN-VGCCs provides an important regulatory mechanism in sustaining the *Ca*^*2*+^ *clock* during normal pacemaking by promoting the *coupled clock*. In addition, our study shows that coupling between Ca_v_1.2 and random LCRs generated by RyR-dependent Ca^2+^ release cannot consistently generate viable SAN automaticity following ablation of SAN-VGCCs and inhibition of f-channels. In this context, our data indicate that future models of pacemaking should include Ca_v_1.3 and Ca_v_3.1 channels as additional mechanisms of coupling between the *Ca*^*2*+^ *clock* and the *membrane clock*, and distinguish between functional roles of Ca_v_1.3 from Ca_v_1.2 channel isoforms.

### Role of SAN-VGCCs in Ca^2+^ entry in SAN diastolic depolarization

Concomitant ablation of SAN-VGCCs abolished *I*_*Ca*_ at voltages spanning the range between ~ − 60 mV and ~ − 35 mV corresponding to diastolic depolarization in wild-type cells (Fig. 1, Supplementary Table 1). Previous work showed that Ca_v_3.1 channels activate at more negative voltages than Ca_v_1.3 channels^7,24^. However, their limited steady-state availability at the maximum diastolic potential (~ 90% steady-state inactivation at − 60 mV)^24^ predicts smaller Ca_v_3.1-mediated *I*_*CaT*_ than Ca_v_1.3-mediated *I*_*CaL*_^23^. This suggests more limited contribution of Ca_v_3.1-mediated *I*_*CaT*_ to SAN diastolic depolarization than of Ca_v_1.3-mediated *I*_*CaL*_. Furthermore, beside the higher steady-state availability of Ca_v_1.3 than Ca_v_3.1 at diastolic voltages, Ca_v_1.3 channels are also an essential molecular component of the sustained inward Na^+^ current (*I*_*st*_)^40^, which constitute an additional contributor to diastolic depolarization^41^. It is thus possible that, while Ca_v_1.3 channels contribute to the pacemaker mechanisms via *I*_*CaL*_/*I*_*st*_ inward currents^24,40^ and generation of LCRs (Ref.^35^ and this study), the predominant contribution of Ca_v_3.1 channels to SAN pacemaking is regulation of LCRs connected to the *coupled clock* mechanism. In line with this hypothesis, we did not observe additivity between individual ablation of Ca_v_1.3 and concomitant ablation of SAN-VGCCs on atrial rate in *vivo* (Fig. 2), as well as in Langendorff-perfused intact hearts (Fig. 3). Thus, another important factor determining similar HRs in *Ca*_*v*_*1.3*^*−/*−^ and *Ca*_*v*_*1.3*^*−/*−^*/Ca*_*v*_*3.1*^*−/*−^ mice is the convergence of SAN-VGCCs to a common downstream intracellular effector: the generation of diastolic LCRs (Fig. 9). This hypothesis is also consistent with previous evidence on the role of Ca_v_3.1-mediated *I*_*CaT*_ in generating diastolic LCRs in atrial subsidiary pacemaker myocytes^37^. However, isolated SAN/atria from *Ca*_*v*_*1.3*^*−/*−^*/Ca*_*v*_*3.1*^*−/*−^ hearts showed significantly slower intrinsic automaticity than that of *Ca*_*v*_*1.3*^*−/*−^ counterparts (Fig. 4). The discrepancy between the additivity of the effect of SAN-VGCCs in isolated *Ca*_*v*_*1.3*^*−/*−^*/Ca*_*v*_*3.1*^*−/*−^ hearts and SAN/atria preparations and the observations in vivo could be explained by the action of the autonomic nervous system and by physiological mechanoelectrical feedback stimulating pacemaking in working hearts in vivo.

### Role of SAN-VGCCs in SAN automaticity

The observation that *Ca*_*v*_*1.3*^*−/*−^*/Ca*_*v*_*3.1*^*−/*−^ hearts ex vivo present with junctional atrial and ventricular rhythmicity rather than SAN rhythm led us to investigate automaticity using isolated SAN/atria preparations. Both and *Ca*_*v*_*3.1*^*−/*−^ and *Ca*_*v*_*1.3*^*−/*−^ presented with shift of the leading pacemaker sites from central to peripheral SAN areas (Fig. 4). Pacemaker shift is a well-known phenomenon in which groups of automatic cells eventually become dominant following manoeuvres that reduce intrinsic spontaneous activity of previously dominant leading sites (see Ref.^42^, for review). Shift is due to heterogeneity of expression of ion channels^42^ and proteins regulating the *Ca*^*2*+^ *clock* in the SAN^43,44^. Recent work has shown that normal SAN impulse is initiated in the leading site by HCN4-expressing cells showing heterogeneous Ca^2+^ signals^45^. Indeed, these cells present with spontaneous LCRs, or LCRs followed by cell-wide Ca^2+^ transients. These cells can entrain the activity of neighbouring cells or cluster of cells. Different clusters can synchronize their activity to promote the emergence of the SAN impulse^45^. It is thus tempting to speculate that deletion of SAN-VGCCs disrupts LCRs in these clusters shifting the pacemaker leading site towards peripheral or extranodal regions that are less sensitive to Ca_v_1.3 and Ca_v_3.1 deletion and LCRs suppression. These secondary pacemaker regions, such as the peripheral SAN or the posterior nodal extension close to the inferior *vena cava*^31^ express high levels of the f-channel protein HCN4, which would constitute an effective backup pacemaker mechanism. This hypothesis is consistent with our observation that, in comparison with *Ca*_*v*_*1.3*^*−/*−^, *Ca*_*v*_*1.3*^*−/*−^*/Ca*_*v*_*3.1*^*−/*−^ SAN-atria preparations more frequently showed alternating impulse initiation in the SAN and in extranodal sites (Fig. 4), and with our previous observations that pacemaker cells of the mouse peripheral SAN show strong HCN4 positive immunostaining^34^. In addition, both cardiac Na_v_1.5 and TTX-sensitive Na_v_ isoforms have been shown to participate to pacemaking in the peripheral SAN and to intranodal conduction in mice^8^ and humans^10^. It is thus possible that peripheral leading sites express elevated densities of *I*_*Na*_ necessary for impulse conduction within the SAN or from the SAN to the atria^10^. Consistent with this hypothesis, ablation of SAN-VGCCs did not affect the SAN-to-atria conduction time (Supplementary Fig. 3). Shift of pacemaker site to peripheral SAN could explain why concomitant inhibition of *I*_*f*_ and TTX-sensitive *I*_*Na*_ arrests automaticity in the majority of *Ca*_*v*_*1.3*^*−/*−^ and *Ca*_*v*_*1.3*^*−/*−^*/Ca*_*v*_*3.1*^*−/*−^ SAN-atria preparations (Fig. 6).

In conclusion, our data show for the first time that SAN-VGCCs are necessary to maintain atrial automaticity in the central SAN region. Concomitant ablation of SAN-VGCCs reduced the HR in vivo by ~ 200 bpm, which corresponds to ~ 30% of the HR measured in wild-type mice under control conditions (Fig. 2). This value is similar to that recorded in wild-type mice under *I*_*f*_ inhibition by ivabradine (~ 200 bpm). However, the HR in some *Ca*_*v*_*1.3*^*−/*−^*/Ca*_*v*_*3.1*^*−/*−^ mice under *I*_*f*_ inhibition was lower than 100 bpm, which corresponds to a reduction of more than 85%, in comparison to the initial HR measured in wild-type mice under control condition. Nevertheless, this residual pacemaking was sufficient to sustain heart function in mice. These data underscore the importance of SAN-VGCCs in the generation of SAN automaticity.

### Role of SAN-VGCCs in impulse conduction and ventricular rhythmicity

We found a clearly additive impact on AV conduction between individual Ca_v_1.3 and concomitant SAN-VGCCs ablation (Figs. 2, 3). More hyperpolarized diastolic voltages in some AVN cells and in myocytes of His’–Purkinje system than in the SAN could explain this additive effect. This would increase the steady-state availability of Ca_v_3.1-mediated *I*_*CaT*_ in the conduction system. Consequently, dissociated rhythms observed in *Ca*_*v*_*1.3*^*−/*−^*/Ca*_*v*_*3.1*^*−/*−^ mice can be explained also by loss of depolarization reserve provided by *I*_*CaT*_ in *Ca*_*v*_*1.3*^*−/*−^ mice. The high incidence of complete heart block with dissociated atrial and ventricular rhythms in *Ca*_*v*_*1.3*^*−/*−^*/Ca*_*v*_*3.1*^*−/*−^ hearts underscores the importance of Ca_v_3.1 channels in atrioventricular conduction (Fig. 3). Shifting of leading pacemaker sites from primary sites to extranodal locations can explain atrial ectopic and atrial rhythmicity observed in Langendorff-perfused *Ca*_*v*_*1.3*^*−/*−^*/Ca*_*v*_*3.1*^*−/*−^ hearts (Supplementary Fig. 2). This impairment of primary SAN automaticity by ablation of SAN-VGCCs could promote ventricular arrhythmia by unmasking the activity of automatic junctional proarrhythmic sites. In particular, complete heart block in *Ca*_*v*_*1.3*^*−/*−^*/Ca*_*v*_*3.1*^*−/*−^ hearts may favor ventricular arrhythmia by unmasking junctional or ventricular ectopic sites. Indeed, we showed previously that episodes of SAN pauses or AV block precede spontaneous initiation of ventricular tachycardia in mice carrying heart specific expression of non-conductive f-channels (HCN4-AYA mice)^34^. A compensatory effect by autonomic tone may explain the absence of ventricular arrhythmia in *Ca*_*v*_*1.3*^*−/*−^*/Ca*_*v*_*3.1*^*−/*−^ mice in vivo. In this respect, we showed that atropine suppressed ventricular tachycardia in HCN4-AYA mice^34^. Thus, blockade of *I*_*KACh*_ activation by atropine could be an additional factor suppressing ventricular arrhythmia in *Ca*_*v*_*1.3*^*−/*−^*/Ca*_*v*_*3.1*^*−/*−^ mice in vivo under inhibition of the autonomic nervous system (Fig. 1). In conclusion, our data show that expression of Ca_v_3.1 channels is essential to maintain normal atrial and ventricular rhythmicity in hearts lacking Ca_v_1.3.

### Role of SAN-VGCCs in the pacemaker mechanism

Beside SAN-VGCCs and *I*_*f*_, other plasmalemmal ion channels, as well as RyR-dependent Ca^2+^ release can contribute to the generation of diastolic depolarization and pacemaker activity. We thus hypothesized that the residual pacemaker activity observed in vivo in *Ca*_*v*_*1.3*^*−/*−^ and *Ca*_*v*_*1.3*^*−/*−^*/Ca*_*v*_*3.1*^*−/*−^ mice or in ex vivo mutant hearts and SAN/atrial preparations under ivabradine was sustained by TTX-sensitive Na_v_1 channels and RyR-dependent Ca^2+^ release.

Previous work has showed that *I*_*f*_ inhibition by ivabradine reduces diastolic LCRs, by slowing the *coupled clock* in a rate-dependent way^46^. In particular, the *coupled clock* model of pacemaking predicts that *I*_*f*_ inhibition will slow pacemaker activity, which in turn, will reduce LCRs via the *coupled clock* mechanism^16,46^. In relation to the present study, it is possible that slowing of diastolic depolarization by *I*_*f*_ inhibition delays activation of SAN-VGCCs contributing to reduction in diastolic LCRs in wild-type SAN cells. However, our data show that deletion of SAN-VGCCs leads already to strong reduction in late diastolic LCRs with consequent disruption of the *coupled clock* mechanism (Fig. 9). The evidence that inhibition of *I*_*f*_ and *I*_*Na*(*TTX*)_ does not reduce the total LCRs number in mutant cells despite further slowing of pacemaking is consistent with our hypothesis that ablation of SAN-VGCCs disrupts the normal functioning of the *coupled clock*. Consequently, it is possible that the role of *I*_*f*_ in residual pacemaker activity observed following concomitant ablation of SAN-VGCCs differs from that observed in wild-type cells, in which the *coupled clock* mechanism is intact before *I*_*f*_ blockade. This hypothesis is in line with our observation that the relative heart rate slowing induced by *I*_*f*_ inhibition is higher in *Ca*_*v*_*1.3*^*−/*−^ and *Ca*_*v*_*1.3*^*−/*−^*/Ca*_*v*_*3.1*^*−/*−^ than in wild-type mice (Fig. 5). This differential contribution of *I*_*f*_ to pacemaking in wild-type and mutant hearts underscores the importance of the depolarization reserve in the intact SAN, in which *I*_*f*_, beside contributing to cellular automaticity, prevents tissue hyperpolarization imposed by atrial electrotonic load^6,47^.

Our data show that inhibition of *I*_*Na(TTX)*_ arrests automaticity in 50% of isolated SAN/atria preparations and in 40% individual *Ca*_*v*_*1.3*^*−/*−^ and *Ca*_*v*_*1.3*^*−/*−^*/Ca*_*v*_*3.1*^*−/*−^ SAN cells. This observation indicates that TTX-sensitive Na_v_1 channels are the predominant mechanisms sustaining pacemaker activity after ablation of SAN-VGCCs and pharmacologic inhibition of *I*_*f*_ (Figs. 6, 7). We may expect that spontaneous RyR-dependent Ca^2+^ release could remain viable under these conditions, as predicted by the *coupled clock* model^3^. Indeed, *Ca*_*v*_*1.3*^*−/*−^ and *Ca*_*v*_*1.3*^*−/*−^*/Ca*_*v*_*3.1*^*−/*−^ hearts express functional Ca_v_1.2 channels and could ensure proper SR Ca^2+^ load to activate spontaneous *Ca*^*2*+^ *clock* mechanism^3^. This hypothesis would be in line with our observation that deletion of Ca_v_1.3 channels does not reduce the SR Ca^2+^ load^27^. However, our data show that SAN-VGCCs are a key factor to generate and control diastolic LCRs underlying the *coupled clock* mechanism of pacemaking. Several combined factors may explain the poor efficiency of Ca_v_1.2 channels in maintaining proper pacemaking, when functionally associated with *I*_*f*_ and spontaneous RyR-dependent Ca^2+^ release. Beside the more positive threshold for activation of Ca_v_1.2 in comparison to Ca_v_1.3 channels, Ca_v_1.3-mediated *I*_*CaL*_ activation kinetics is faster and inactivation is lower than that of Ca_v_1.2-mediated *I*_*CaL*_ in SAN cells^24^. Differences in channels’ kinetics probably render Ca_v_1.3 more adaptable to generation of rhythmic activity in SAN cells than Ca_v_1.2 channels. Moreover, the membrane distribution of Ca_v_1.2 channels differs from that of Ca_v_1.3 in mouse SAN cells. Indeed, we showed previously that while Ca_v_1.2 immunoreactivity is evenly distributed along the plasma membrane, Ca_v_1.3 immunoreactivity shows well-defined co-staining with RyR2^48^. This differential subcellular distribution of Ca_v_1.3 channels may facilitate control of LCRs in the diastolic phase in comparison to Ca_v_1.2 channels that reside in a less-favorable spatial position to generate LCRs. In line with this hypothesis, we previously showed that ablation of Ca_v_1.3 channels abolishes synchronous recruitment of diastolic RyR-dependent Ca^2+^ releasing sites under adrenergic activation of pacemaker activity^27^. Loss of synchronous recruitment intervenes despite the significant negative shift in the threshold for activation of Ca_v_1.2-mediated *I*_*CaL*_ induced by adrenergic activation (~ 5 mV)^27^.

It is possible that residual RyR-dependent Ca^2+^ release was effective in the fraction of SAN/atria preparations still generating automaticity at very low rates, probably in cooperation with TRPC^14^ and TRPM4^12^ channels. Nevertheless, our results show that expression of SAN-VGCCs is necessary to ensure pacemaking, via Ca^2+^ entry and efficient generation of diastolic LCRs in diastolic depolarization; two functions that sole expression of Ca_v_1.2 channels could not facilitate.

## Conclusions

Our study demonstrates that co-expression of functional Ca_v_1.3 and Ca_v_3.1 SAN-VGCCs is essential for generating normal heart automaticity and atrioventricular conduction, as well as to stabilize atrial and ventricular rhythmicity and generating SAN pacemaker activity. We show that normal heart automaticity is reliant on expression of SAN-VGCCs to generate diastolic inward current and Ca^2+^ entry to activate the *Ca*^*2*+^ *clock* pacemaker mechanism during normal pacemaking. As a corollary, our study indicates that functional association of L-type Ca_v_1.2 channels and f-channels is not sufficient to maintain normal SAN pacemaking and heart automaticity. Finally, our study provides new mechanistic insight into congenital and autoimmune SAN dysfunction and atrioventricular block based on SAN-VGCC loss-of-function^29,30,49^.

## Methods

Mutant mice harboring genetic ablation of VGCCs (*Ca*_*v*_*3.1*^*−/*−^, *Ca*_*v*_*1.3*^*−/*−^ and *Ca*_*v*_*1.3*^*−/*−^*/Ca*_*v*_*3.1*^*−/*−^*)* were generated in the IGF animal facility from C57B/6J genetic background. *Ca*_*v*_*1.3*^*−/*−^*/Ca*_*v*_*3.1*^*−/*−^ mice were obtained by crossing C57B/6J *Ca*_*v*_*1.3*^*−/− *27^ with C57B/6J *Ca*_*v*_*3.1*^*−/−*^^25^ mice. The investigation conforms to the Guide for the Care and Use of Laboratory Animals published by the US national Institute of Health (NIH Publication No. 85–23, revised 1996) and European directives (2010/63/EU). The experimental procedure was approved by the Ethical committee of the University of Montpellier and the French Ministry of agriculture (protocol no: 2017010310594939). Animals were housed in individual cages with free access to food and water and were exposed to 12-h light/dark reverse cycles (light, 20:00 h to 8:00 h) in a thermostatically controlled room.

### ECG recordings in mice

Telemetric ECG recordings in freely-moving mice were performed as previously described^23,34,35^. Briefly, mice were anesthetized using gas anesthesia with 2% isoflurane (Forene, Abbott, UK). An ETA-F10 telemetric transmitter (Data Sciences International) was placed subcutaneously along the animal’s back. Transmitter’s wire electrodes were placed in DII derivation against the heart axis. Advil (ibuprofen, 7 mL/L) was added to the drinking water for 4 days after implantation to prevent post-operative pain. Mice were left to recover for 10 days before experiments. ECG signals were recorded by employing a telemetric receiver connected to an analog-to-digital conversion acquisition system. Analysis was performed off-line using the Dataquest A.R.T. software (Data Sciences International). Heart rates (HR) were determined from RR intervals and atrial rates from PP intervals, under control conditions or following recording of 4-h baseline to evaluate drugs effects. All drugs were administered by intraperitoneal (IP) injection. Mean HR values were calculated by analyzing periods of 5 min at different time points corresponding to the peak effect of the drug. ECG parameters were measured by employing ECG Auto 1.5.7 software (EMKA Technologies).

### Langendorff-perfused hearts

ECG recordings in Langendorff perfused mouse hearts were performed as previously described^35^. Mice were deeply anesthetized by IP injection of 0.3 mL of solution constituting Ketamine (0.1 mg/g, Imalgène) and Xylazine (0.01 mg/g, Rompun 2%, Bayer), followed by a second injection of Pentobarbital (150 µL Euthasol Vet in 10 mL NaCl physiological solution). To avoid blood clots in the heart, we injected 0.5 mL of NaCl solution containing Heparin (25,000 U.I). Hearts were removed via thoracotomy when the tail sensitivity test become negatives. Excised hearts with aortic cannula were quickly mounted on a Langendorff apparatus (Isolated heart system; EMKA Technologies) at a pressure of 70–80 mm Hg imposed by the working heart with normal Tyrode’s solution containing (mM): NaCl, 140; KCl, 5.4; MgCl_2_, 1; CaCl_2_, 1.8; Hepes, 5 and glucose, 5.5 (pH adjusted to 7.4 with NaOH). Perfused hearts were immersed in the water-jacked bath and maintained at 36 °C. The ECG was continuously recorded by two Ag–AgCl electrodes; the first was placed on the epicardial side of the right atrium close to the SAN area and the second near the ventricular apex. The heart rate was allowed to stabilize for at least 30 min before perfusion of drugs. ECG parameters were measured with ECG Auto 3.3.3.8 software (EMKA Technologies).

### Intact SAN/atria preparations

We obtained SAN-atria preparations from excised hearts as previously described^50^. Briefly, we placed the entire SAN/atrial preparation in pre-warmed (36 °C) Tyrode’s solution containing heparin (10 U/mL). We used a stereomicroscope (SZX16; Olympus) with low magnification (7 ×) to transilluminate and visualize directly the isolated SAN/atria preparation. We identified the SAN region using the superior and inferior vena cava, the *crista terminalis*, and the interatrial septum as landmarks. The SAN/atrial preparation including right and left atria (RA and LA) was pinned to the bottom of an optical chamber (Fluorodish, FD35PDL-100; WPI) coated with ~ 2 mm of clear Sylgard (Sylgard 184 Silicone elastomer kit; Dow Corning). To maintain the SAN in a flat plane, we pinned the right and left atrium using entomology needles.

### Optical mapping of membrane voltage in SAN/atria preparations

Optical mapping of SAN impulse was performed as described previously^51^. SAN/atria preparation was loaded with the voltage-sensitive dye indicator Di-4-ANEPPS in Tyrode’s solution with 10 μM Biotium for at least 30 min at room temperature (20–22 °C). The preparation was placed on an agitated plate to maintain proper oxygenation of the tissue and load it uniformly. The tissue was then washed 3 times in dye-free Tyrode’s solution. The SAN/atria preparation was continously perfused at 34–36 °C. A MICAM Ultima-L complementary metal oxide semiconductor (CMOS) camera (100 × 100-pixel CMOS sensor, 10 × 10 mm, SciMedia) was used to achieve high speed optical mapping of membrane voltage (2 ms/frame). The camera was mounted on a THT microscope, with two objectives (2 × and 1.6 ×) to generate a field view of 12.5 × 12.5 mm. The excitation light source consisted in a 150-W halogen light system with built-in shutter (SciMedia). The filter set included a 531/50-nm excitation filter, 580-nm dichroic mirror, and 580 long-pass emission filter. Blebbistatin (1.5–5 μM; Tocris Bioscience) was used to prevent imaging artifacts due to tissue contraction. We usually limited our recording times to 32.768 s (16,384 frames at 2 ms per frame) to preserve the tissue from dye phototoxicity. Data were analyzed using the BrainVision Analyzer software (BrainVision).

### Isolation of SAN cells

SAN pacemaker cells were isolated as previously described^52^. Briefly, SAN tissue was immersed into solution containing 140 mM NaCl, 5.4 mM KCl, 0.5 mM MgCl_2_, 0.2 mM CaCl_2_, 1.2 mM KH_2_PO_4_, 50 mM taurine, 5.5 mM D-glucose, 1 mg/mL BSA, and 5 mM Hepes–NaOH (adjusted to pH 6.9 with NaOH) for 4 min. The tissue was then transferred into the low-Ca^2+^ solution containing purified collagenase and protease (Liberase medium Thermolysin concentration; 229 U/mL; Roche). 1.9 U/mL elastase (Boehringer Mannheim). Digestion was carried out for 15–20 min at 36 °C. To stop the digestion process, the SAN was washed in a medium containing 70 mM l-glutamic acid, 20 mM KCl, 80 mM KOH, 10 mM KH_2_PO_4_, 10 mM taurine, 1 mg/mL BSA, and 10 mM Hepes–KOH (adjusted to pH 7.4 with KOH). Single cells were isolated from the SAN tissue by manual agitation using a flame-forged Pasteur’s pipette in KB solution at 36 °C. To recover the automaticity of the SAN cells, Ca^2+^ was gradually reintroduced in the cells’ storage solution to a final concentration of 1.8 mM^52^.

### Patch-clamp recordings of SAN cells

Patch-clamp recordings of SAN cells was carried out as previously described^52^. Cells were harvested in custom-made chambers with glass bottoms for cell attachment. Cells were then superfused with Tyrode’s solution warmed at 36 °C before recording. Voltage-gated Ca^2+^ currents (*I*_*Ca*_) were recorded using standard whole cell patch-clamp configuration as previously described^23,24^. Extracellular recording solution contained (mM): 135 tetraethylammonium chloride (TEA-Cl), 10 4-aminopyridine (4-AP), 1 MgCl_2_, 0.03 tetrodotoxin (TTX), 1 g/L Glucose, 2 CaCl_2_, 10 Hepes, (adjusted to pH = 7.2 with TEAOH). Electrodes had a resistance of 3 MΩ and were filled with a solution containing (in mM): 125 Cs^+^-aspartate, 20 TEA-Cl, 1.2 CaCl_2_, 5 Mg-ATP, 0.1 Li_2_-GTP, 5 EGTA, and 10 HEPES (pH adjusted to 7.2 with CsOH)^23,24^. Seal resistances were in the range of 2–5 GΩ. Pacemaker activity of SAN cells was recorded under perforated patch conditions by adding 50 μM β-Escin to the pipette intracellular solution^23,35^. Patch-clamp electrodes had a resistance of 3–5 MΩ when filled with an intracellular solution containing (in mM): 130 K^+^-aspartate, 10.0 NaCl; 2 ATP-Na^+^ salt, 6.6 creatine phosphate, 0.1 GTP-Mg^2+^, 0.04 CaCl_2_ (pCa = 7.0), and 10.0 HEPES–KOH (adjusted to pH 7.2 with KOH). Perfusion of pre-warmed (36 °C) experimental solutions was performed by using a multi-MPRE8 heating pen (Cell Micro Controls,). Data acquisition was performed using a Multiclamp 700A patch-clamp amplifier connected to Digidata 1550B interface (Molecular Devices).

### Ca^2+^ imaging in isolated SAN cells

Confocal Ca^2+^ imaging of SAN cells was performed as previously described^27^. SAN cells were harvested in a glass Fluorodish (FD3510-100, WPI), coated overnight with laminin (1 mg/mL; Sigma-Aldrich) for 1 h before recordings. SAN cells loading was performed by removing the bath solution and replacing it by Tyrode’s solution containing the Ca^2+^ indicator CAL-520 (1 µM, from a stock solution containing DMSO/Pluronic F-127 0.13%; Invitrogen) during 25 min at room temperature. Images were obtained with confocal microscopy (Zeiss LSM 780), by scanning SAN cells with an Argon laser in line-scan configuration. To avoid phototoxicity we generally recorded the SAN cells at a line rate of 1.53 or 3.78 ms to obtain 10,000 lines. Fluorescence was excited at 488 nm and emissions were collected at > 505 nm. A 63 × oil immersion objective was used to record [Ca^2+^]_i_ in isolated SAN myocytes. We suppressed the background noise and analyzed the time-courses of Ca^2+^ fluorescence by pClamp software (ver.10.6.2.2, Molecular Devices). [Ca^2+^]_i_ transients and LCRs were analyzed using ImageJ software 8. To identify the ramp phase of the [Ca^2+^]_i_ transient, we integrated the F/F_0_ signal 150 ms before the peak of the cell-wide transient and calculated the slope of this phase using Clampfit (ver. 10.7).

### Statistical analysis

Statistical analysis was performed using Prism 8.0 (GraphPad Software). Data are represented as mean ± the standard error of the mean (SEM) or the standard error (SD) as indicated. Statistical tests used in each experiment are specified throughout the figure legends. Statistical significance was defined as: *p < 0.05, **p < 0.05, p < 0.01, ***p < 0.001 and ****p < 0.0001.

## Supplementary information

Supplementary Information.
