# Supplementary material for: Concomitant genetic ablation of L-type Cav1.3 (α1D) and T-type Cav3.1 (α1G) Ca2+ channels disrupts heart automaticity
Source: Sci Rep. 2020 Nov 3;10:18906. doi: 10.1038/s41598-020-76049-7 (PMC7642305; doi:10.1038/s41598-020-76049-7)
Supplement: Supplementary file 1 — Supplementary Information. [file 41598_2020_76049_MOESM1_ESM.pdf]

Supplementary Online Section

Concomitant genetic ablation of L-type $Ca_v1.3$ (α_{1D}) and T-type $Ca_v3.1$ (α_{1G}) Ca^{2+} channels disrupts heart automaticity.

M. Baudot[1,2], E. Torre[1,2,3], I. Bidaud[1,2], A.G. Torrente[1,2], J. Louradour[1,2] L. Fossier[1,2], L. Talssi[1,2], J. Nargeot[1,2], S. Barrère-Lemaire[1,2], P. Mesirca[1,2], M.E. Mangoni[1,2]

[1]Institut de Génomique Fonctionnelle, Université de Montpellier, CNRS, Inserm, Montpellier, France.

[2]LabEx ICST, Montpellier, France.

[3]Department of Biotechnology and Biosciences, Università degli Studi di Milano-Bicocca, Milan, Italy.

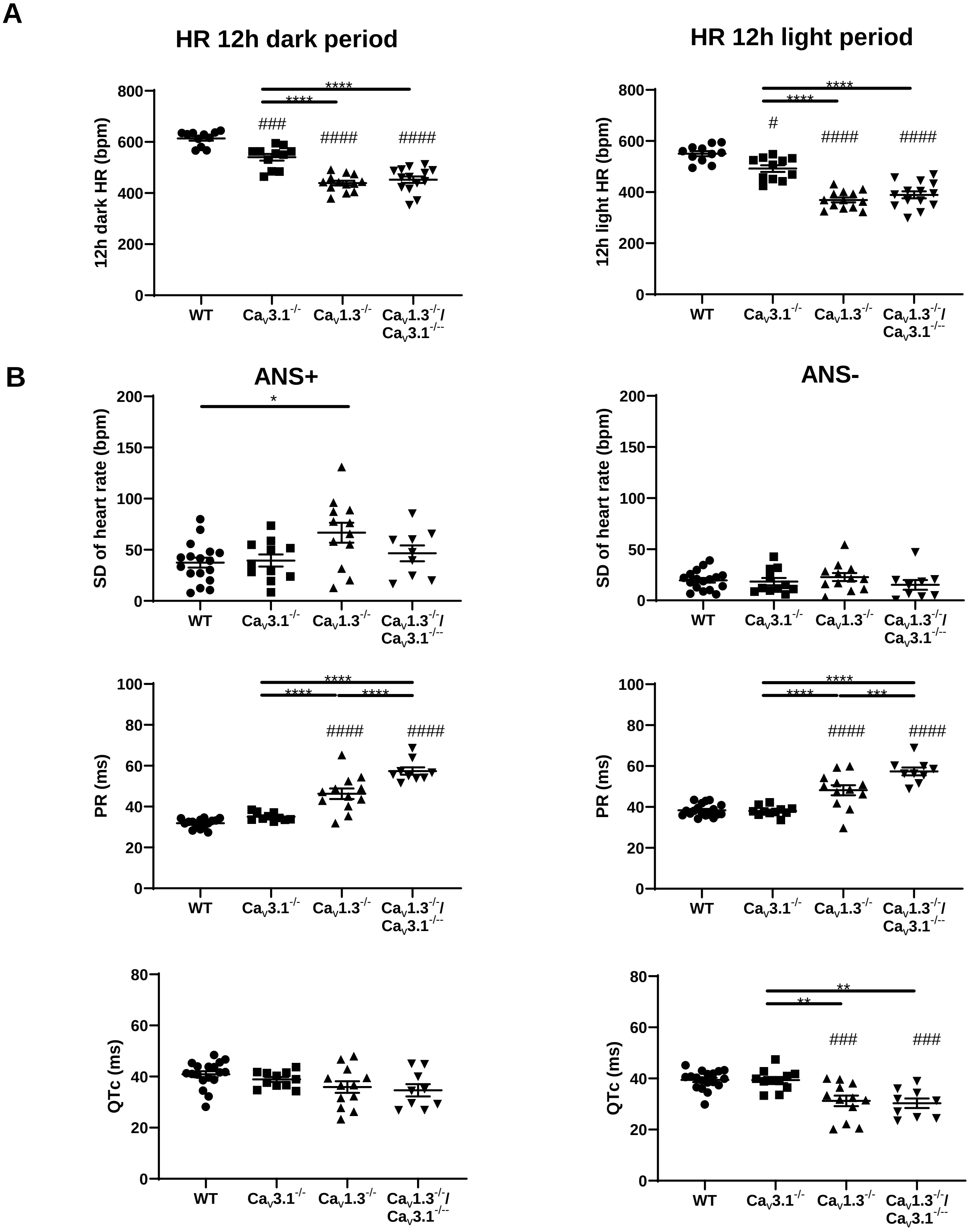

Supplementary Fig.1. ECG in wild-type and mutant mice during 12h dark (left) and 12h light (right) period.. Wild-type (*WT*) n=11, $Ca_v3.1^{-/-}$ n=11, $Ca_v1.3^{-/-}$ n=13, $Ca_v1.3^{-/-}/Ca_v3.1^{-/-}$ n=14 (A). Standard deviation of the heart rate (top), averaged PR (center) and QT (bottom) intervals under basal conditions (ANS+) and after pharmacologic inhibition of the autonomic nervous system (ANS-). Wild-type (*WT*) n=17, $Ca_v3.1^{-/-}$ n=11, $Ca_v1.3^{-/-}$ n=12, $Ca_v1.3^{-/-}/Ca_v3.1^{-/-}$ n=9. Statistics: one-way ANOVA followed by Tukey's multiple comparisons test. (#) indicates comparison with WT.

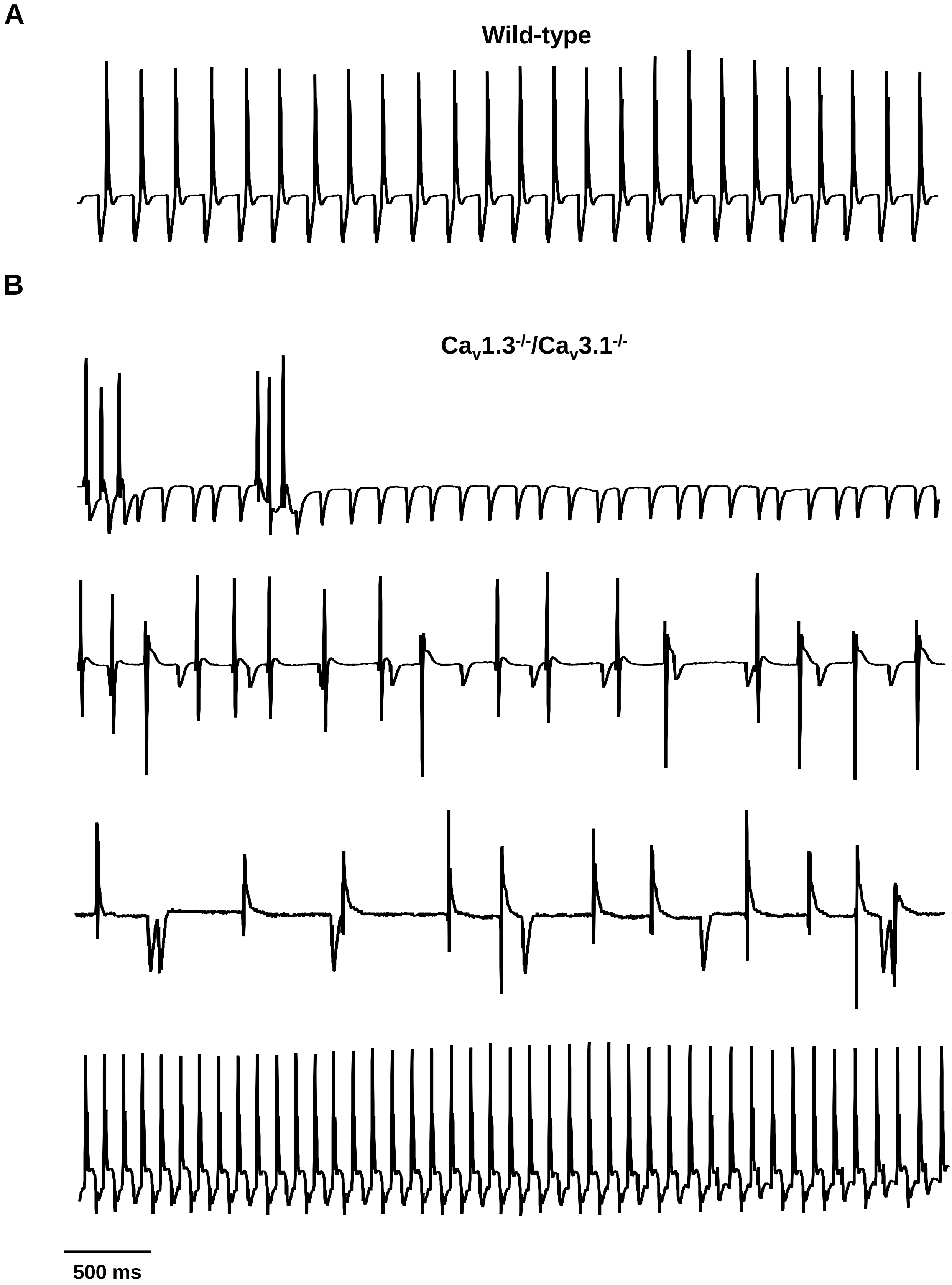

Supplementary Fig.2. Examples of ECGs from isolated heart. Sample ECGs recordings from Langendorff perfused wild-type *(WT)* hearts (A) and hearts from $Ca_v1.3^{-/-}/Ca_v3.1^{-/-}$ mice (B) showing atrial and ventricular arrhythmias, including premature ventricular contraction, atrioventricular blocks and ventricular tachycardia.

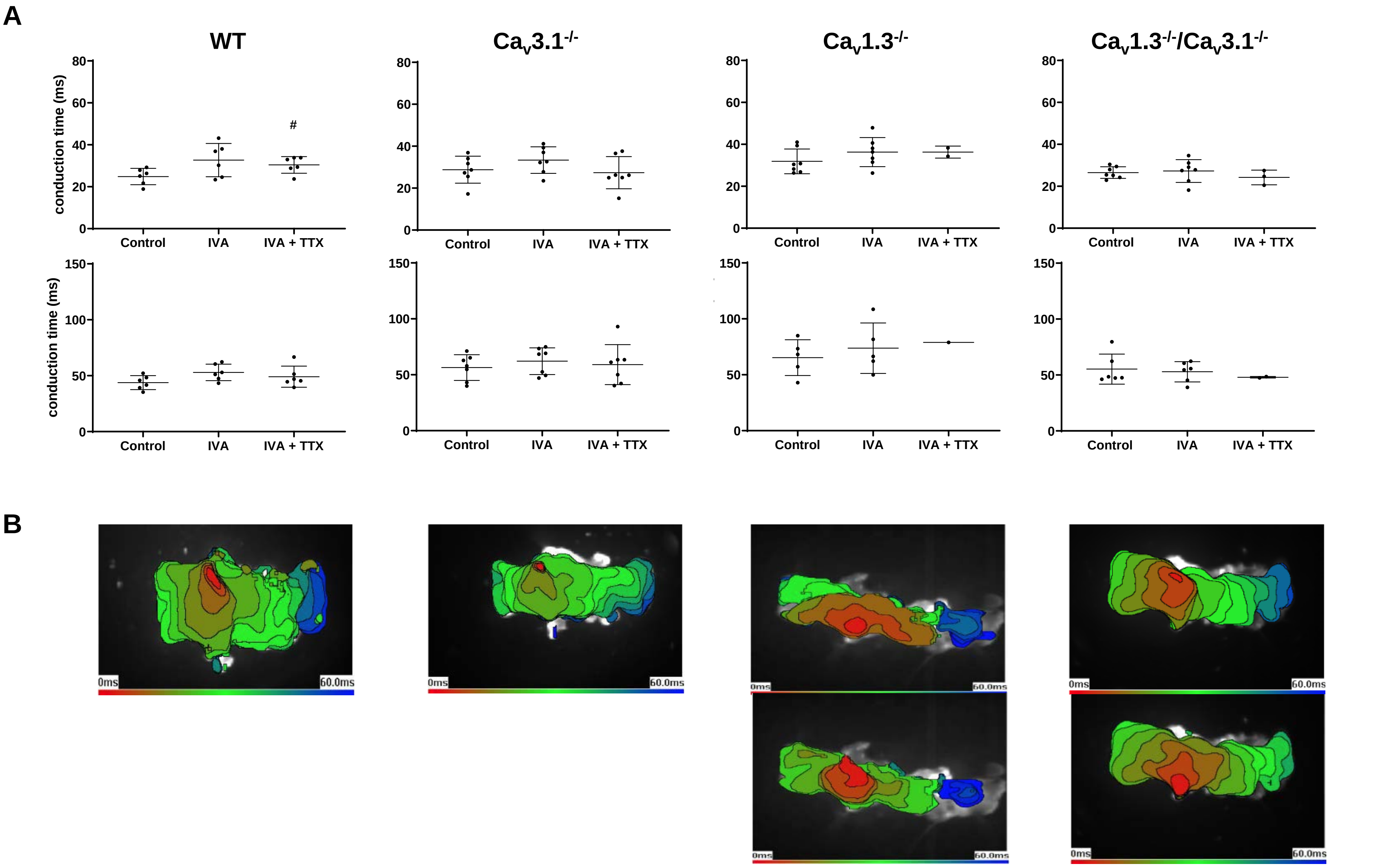

Supplementary Fig.3. Conduction time in SAN/atria preparations. (A) Conduction time between the leading site toward right (top) and left (bottom) atria calculated in tissues from different mutants perfused with tyrode solution (control), with 10 µM ivabradine or with 10 µM ivabradine plus 100 nM TTX. *WT* n=6, *Ca$_v$3.1$^{-/-}$* n=7, *Ca$_v$1.3$^{-/-}$* n=7, *Ca$_v$1.3$^{-/-}$/Ca$_v$3.1$^{-/-}$* n=7. Statistics: One-way ANOVA followed by Tukey's test. (B) Leading region position in SAN/atria preparations. SAN/atria tissue activation maps isolated from different mouse strains (color gradient, 4 ms/color). Multiple leading regions are recorded in *Ca$_v$1.3$^{-/-}$* and *Ca$_v$1.3$^{-/-}$/Ca$_v$3.1$^{-/-}$* SAN/atria preparations (bottom).

Supplementary Table 1. Action potential (AP) parameters recorded in isolated SAN cells from wild-type (*WT*), *Ca*$_v$*3.1*$^{-/-}$, *Ca*$_v$*1.3*$^{-/-}$ and *Ca*$_v$*1.3*$^{-/-}$/*Ca*$_v$*3.1*$^{-/-}$ mice in Tyrode's solution. The top panel shows a sample segment of an AP recording with time- and voltage-hallmarks used for AP analysis. MDP: maximal diastolic potential (mV); Eth: AP threshold (mV); APA: AP amplitude (mV); APD: AP duration (ms); SLDD: slope of the linear part of the diastolic depolarization (mV/ms); EDD: slope of the exponential part of the diastolic depolarization (mV/ms); dV/dt: AP upstroke (mV/ms). Statistics: one-way ANOVA followed by Tukey's multiple comparisons test. Data are represented as mean±SEM, with P values.

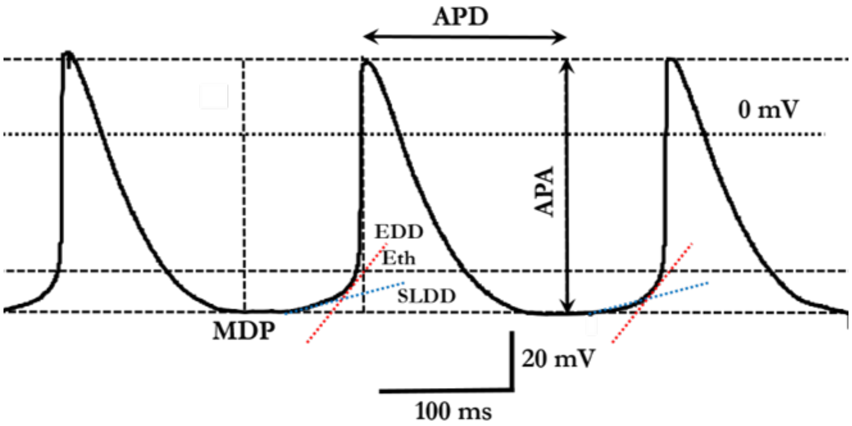

	WT (A) n=14	*Ca*$_v$*3.1*$^{-/-}$ (B) n=17	*Ca*$_v$*1.3*$^{-/-}$ (C) n=11	*Ca*$_v$*1.3*$^{-/-}$ /*Ca*$_v$*3.1*$^{-/-}$ (D) n=13	P A vs B	P A vs C	P A vs D	P B vs C	P B vs D	P C vs D
**MDP (mV)**	**-62±2**	**-61±2**	**-61±1**	**-63±1**	**0.9684**	**0.9932**	**0.9905**	**0.9983**	**0.8775**	**0.9462**
**Eth (mV)**	**-37±1**	**-37±1**	**-42±1**	**-45±1**	**0.9963**	**0.0169**	**<0.0001**	**0.0155**	**<0.0001**	**0.5157**
**APA (mV)**	**91±3**	**93±4**	**81±12**	**90±5**	**0.9921**	**0.6844**	**>0.9999**	**0.5605**	**0.9885**	**0.6923**
**APD (ms)**	**150±16**	**156±9**	**199±24**	**194±13**	**0.9920**	**0.1657**	**0.1456**	**0.3170**	**0.3188**	**0.9966**
**SLDD (mV/ms)**	**0.11±0.02**	**0.08±0.02**	**0.02±0.01**	**0.02±0.01**	**0.4450**	**<0.0001**	**<0.0001**	**0.0201**	**0.0036**	**0.9942**
**EDD (mV/ms)**	**1.0±0.2**	**0.8±0.1**	**0.3±0.1**	**0.2±0.1**	**0.5904**	**0.0002**	**<0.0001**	**0.0191**	**0.0023**	**0.9828**
**dV/dt (mV/ms)**	**39±7**	**39±7**	**31±5**	**26±4**	**>0.9999**	**0.8082**	**0.3429**	**0.8335**	**0.4016**	**0.9240**

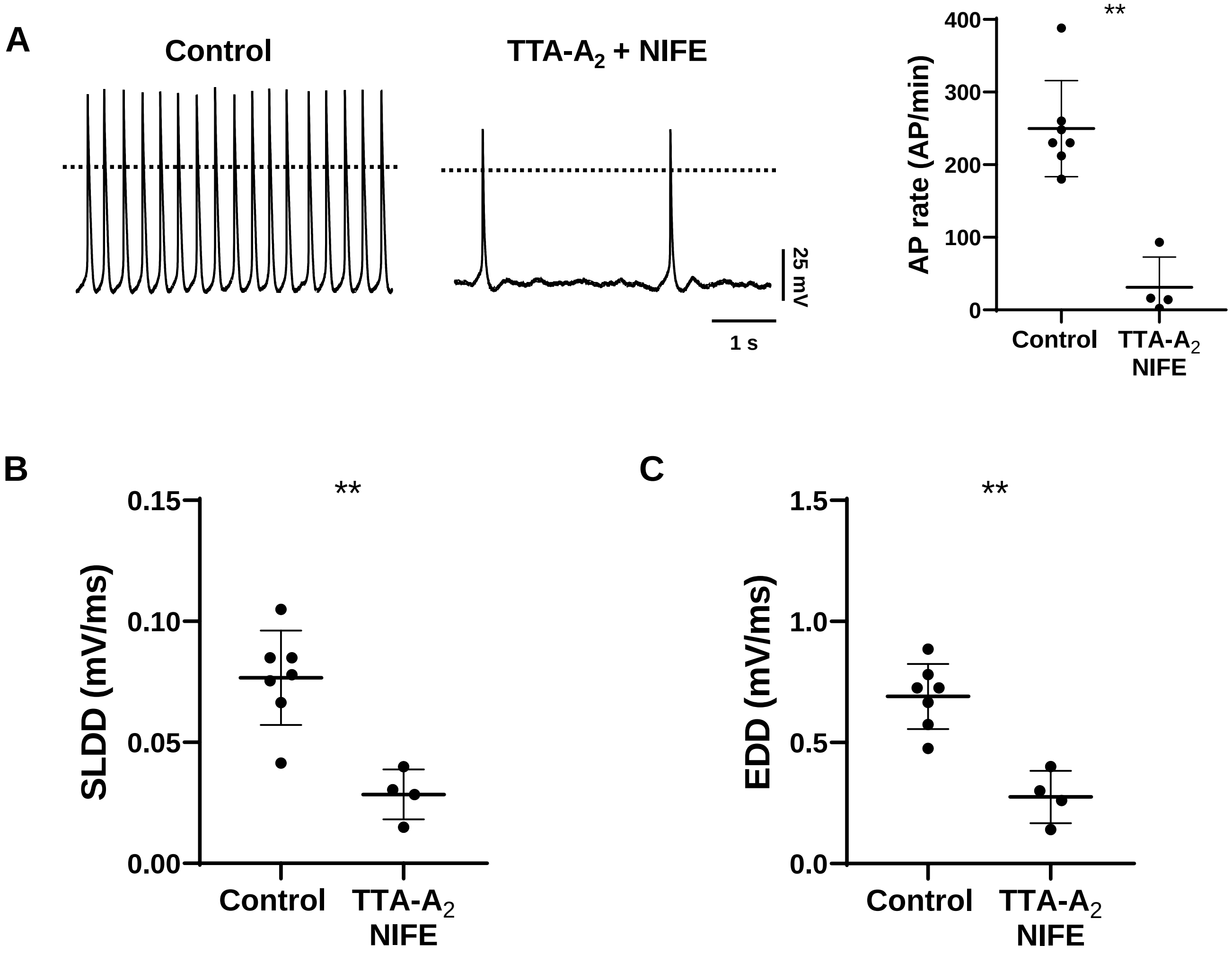

Supplementary Fig. 4. Automaticity in isolated *Cav1.2*$^{DHP-/-}$ SAN cells perfused with T- and L-type Ca^{2+} channel blockers. (A) Samples of perforated-patch action potential recordings and averaged action potential rates from *Cav1.2*$^{DHP-/-}$ SAN cells in control conditions (n=7) and under concomitant perfusion of 1 μM TTA-A_2 and 3μM nifedipine (n=4). Slope of the linear part (B) and of the exponential part (C) of the diastolic depolarization phase recorded in *Cav1.2*$^{DHP-/-}$ SAN isolated cells in control (n=7) and after perfusion of 1μM TTA-A_2 and 3μM nifedipine (n=4). Statistics: Mann-Whitney-test. Whiskers indicate mean ± the SD.

Supplementary Table 2. Maximal diastolic potential (MDP) recorded in isolated SAN cells from wild-type (*WT*), *$Ca_v3.1^{-/-}$*, *$Ca_v1.3^{-/-}$* and *$Ca_v1.3^{-/-}$/$Ca_v3.1^{-/-}$* mice in Tyrode's solution and after perfusion of 3μM ivabradine. Statistics: paired t-test. Data are represented as mean±SEM.

	**MDP (mV)** Tyr	**MDP (mV)** IVA	**p value**
**WT** n=11	**-62±1**	**-64±1**	**0.0250**
$Ca_v3.1^{-/-}$ n=17	**-61±1**	**-63±1**	**0.0093**
$Ca_v1.3^{-/-}$ n=10	**-61±1**	**-65±2**	**0.0099**
$Ca_v1.3^{-/-}$/$Ca_v3.1^{-/-}$ n=8	**-65±2**	**-66±2**	**0.6146**

Supplementary Table 3. Action potential (AP) parameters recorded in isolated SAN cells from *$Ca_v1.2^{DHP-/-}$* mice. AP parameters calculated in pacemaker cells from *$Ca_v1.2^{DHP-/-}$* mice in control condition (Ctrl, Tyrode's solution) and after perfusion of 1 μM TTA-A_2 and 3μM nifedipine. MDP: maximal diastolic potential (mV); Eth: AP threshold (mV); APA: AP amplitude (mV); APD: AP duration (ms); SLDD: slope of the linear part of the diastolic depolarization (mV/ms); EDD: slope of the exponential part of the diastolic depolarization (mV/ms); dV/dt: AP upstroke (mV/ms). Statistics: Mann-Whitney test. Data are represented as mean±SEM.

	**Ctrl n=7**	**TTA-A_2+NIFE n=4**
**MDP (mV)**	**-56±1**	**-56±2**
**Eth (mV)**	**-36±1**	**-39±3**
**APA (mV)**	**87±3**	**84±6**
**APD (ms)**	**120±8**	**148±15**
**SLDD (mV/ms)**	**0.08±0.01**	**0.03±0.01****
**EDD (mV/ms)**	**0.7±0.1**	**0.3±0.1****
**dV/dt (mV/ms)**	**41±5**	**23±5**

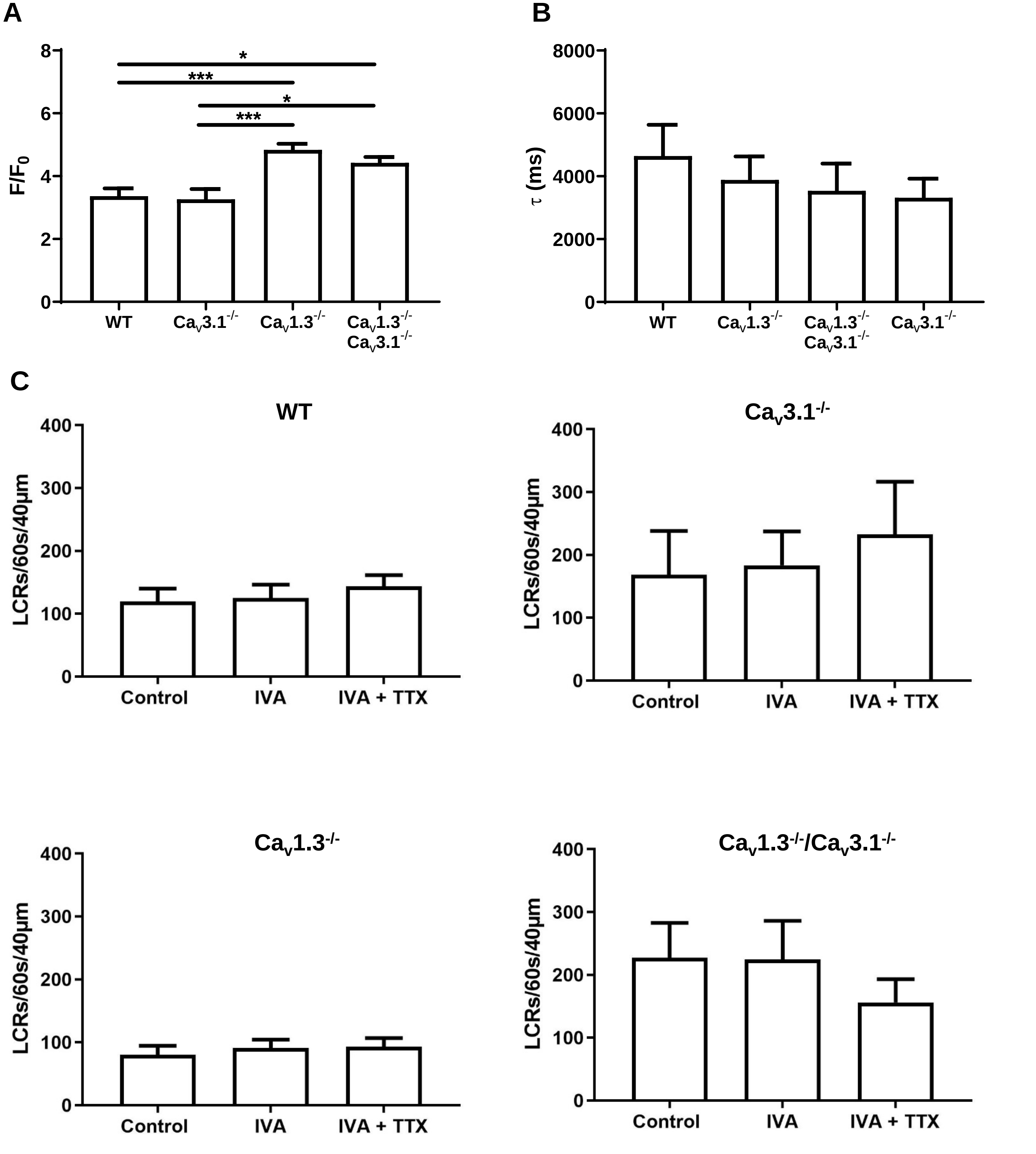

Supplementary Fig.5. Histograms of caffeine-evoked (10 mM) Ca^{2+} release (A, *WT* n=9, $Ca_v3.1^{-/-}$ n=8, $Ca_v1.3^{-/-}$ n=11 and $Ca_v1.3^{-/-}/Ca_v3.1^{-/-}$ n=7) and decay (B, *WT* n=4, $Ca_v3.1^{-/-}$ n=6, $Ca_v1.3^{-/-}$ n=7 and $Ca_v1.3^{-/-}/Ca_v3.1^{-/-}$ n=5) in SAN cells. Statistics: One-way ANOVA followed by Tukey multicomparison test. (C). Frequency of local Ca^{2+} release (LCRs) in SAN cells recorded in pacemaker cells from *WT* (n=11), $Ca_v3.1^{-/-}$ (n=7), $Ca_v1.3^{-/-}$ (n=12) and $Ca_v1.3^{-/-}/Ca_v3.1^{-/-}$ (n=7) in control condition and after perfusion of 3µM ivabradine or 3µM ivabradine plus 50 nM TTX. Statistics: One-way ANOVA followed by Holm-Sidak multicomparison test.
